# Mathematical modeling of control strategies for the elimination of soil-transmitted helminthiases in Thailand

**DOI:** 10.1371/journal.pntd.0013435

**Published:** 2025-08-22

**Authors:** Pavadee Chuaicharoen, Oranard Wattanawong, Dorn Watthanakulpanich, Chawarat Rotejanaprasert, Sopon Iamsirithaworn, Poom Adisakwattana, Wirichada Pan-ngum

**Affiliations:** 1 Department of Tropical Hygiene, Faculty of Tropical Medicine, Mahidol University, Bangkok, Thailand; 2 Ministry of Public Health, Nonthaburi, Thailand; 3 Department of Helminthology, Faculty of Tropical Medicine, Mahidol University, Bangkok, Thailand; 4 Mahidol-Oxford Tropical Medicine Research Unit, Faculty of Tropical Medicine, Mahidol University, Bangkok, Thailand; Wageningen UR: Wageningen University & Research, NETHERLANDS, KINGDOM OF THE

## Abstract

Soil-transmitted helminthiases, caused by soil-transmitted helminths (commonly known as intestinal worms), are considered to be neglected tropical diseases (NTDs). In Thailand, school-age children (SAC) who live in remote areas are at risk of STH. A school-based combined intervention involving test and treat (TnT) and mass drug administration (MDA) programs has been implemented as part of the national STH control program since 2002, with a target to eliminate STH in SAC by 2026. To help achieve this target, we developed an STH transmission dynamic model, calibrated it using STH infection prevalence data from Thailand, and used the model to simulate the effects of the current intervention targeting only SAC and expanded interventions that also cover preschool-age children (PSAC) and adults. We also investigated modified interventions, including biannual MDA and TnT. Our model predicted that neither the current nor a biannual TnT can achieve elimination, even with expanded target populations. However, all biannual MDA treatment scenarios showed a reduced prevalence of STH in SAC, of less than 5%, by 2026. Our model also predicted that biannual MDA targeting SAC and adults would be more effective than targeting SAC and PSAC. Our findings suggest that if community-wide biannual MDA treatment were to be included in the control program, this would be beneficial for eliminating STH in Thailand.

## Author summary

Soil-transmitted helminths (STH), commonly known as intestinal worms, are responsible for diseases known as soil-transmitted helminthiases. As part of annual STH control program activities in Thailand, test and treat (TnT) followed by mass drug administration (MDA) within 6 months, have been implemented among school-age children (SAC), with the aim of alignment with the global campaign to eliminate STH. Although this routine annual control strategy has reduced the prevalence of STH infections in many settings in Thailand, the reduction in prevalence has slowed, putting the goal of reducing prevalence levels to less than 5% by 2026 at risk. New strategies are therefore needed. We developed a mathematical model to explore different control programs that could help to achieve Thailand’s STH elimination goals. Our model predicted that by continuing to deploy the current control program or switching to biannual TnT, we would not reach the 2026 target. However, the target could be achieved with biannual MDA, especially if MDA is to be administered at least among adults as well as school-age children. These findings provide useful guidance for developing appropriate control strategies for eliminating STH in Thailand.

## Introduction

Soil-transmitted helminthiases are infections caused by parasites known as nematode helminths and are considered to be neglected tropical diseases (NTDs) [1,2]. These diseases are caused by infections with one of four species of nematode helminths: *Ascaris lumbricoides* (roundworm), *Trichuris trichiura* (whipworm), and *Ancylostoma duodenale* and *Necator americanus* (hookworms). Soil-transmitted helminthiases contribute to the greatest number of disability-adjusted life years (DALYs) among helminth infections, which also include schistosomiasis, lymphatic filariasis, and onchocerciasis [3]. Typical clinical symptoms include anemia, malnutrition, abdominal pain, diarrhea, and impaired cognitive development [4]. The World Health Organization (WHO) set a target to control STH by 2020, aiming for STH treatment coverage of 75% among preschool-age children (PSAC) and school-age children (SAC) in any endemic area [1]. The number of DALYs lost due to STH was estimated to be 1.9 million in 2019 (a 53% reduction compared with the number of DALYs lost in 2000). This reduction in DALYs occurred in parallel with the increase in coverage of control programs around the world [5]. In 2021, there were an estimated 642.72 million cases and 1.38 million DALYs caused by STH infections. Among STH infections, ascariasis affected 293.80 million cases, resulting in 647.53 thousand DALYs. Additionally, it was estimated to have caused a total of 3,472 deaths [6]. STH are targeted for elimination as a public health problem by 2030. STH control programs worldwide are focused on reducing moderate- and heavy-intensity STH in PSAC and SAC to less than 2% [7]. STH control strategies primarily target children who are at high risk of these infections. WHO recommends three approaches to control and eliminate STH. First, preventive chemotherapy through mass drug administration (MDA), which involves administering anthelmintic drugs at regular intervals regardless of an individual’s infection status; second, promotion of health and hygiene education; and third, improvement of water and sanitation facilities [1]. MDA is considered one of the most important tools available for controlling STH.

In Thailand, according to data from the Thai Ministry of Public Health, STH persist among SAC, especially in rural areas. The national control program for helminth infections in Thai children is being implemented since 2002. The 10-year strategic plan (2017 – 2026) for controlling helminth infection, with annual targets established for monitoring progress has been implemented in Thailand [8]. During this study, Thai government aimed to reduce STH prevalence to below 5% by 2026. This national effort aligns with the World Health Organization’s (WHO) global strategy, which seeks to mitigate the severity of infections and reduce prevalence by 2030. Current intervention strategies targeting SAC include test and treat (TnT) interventions and MDA with albendazole (ALB) [8]. The Kato-Katz (KK) diagnostic technique for the detection of helminth eggs in stools is employed to assess children’s helminth infection status. Children who test positive are then given the appropriate treatment based on their specific helminth infections. TnT usually starts in June each year, followed by MDA six months later. Despite the effort, the prevalence of STH in SAC has remained stable at 5% in 2015–2020 [8]. Although Thailand is transitioning toward the pre-elimination and elimination phases, a persistence of infections in some populations potentially posing new challenges for the design of an optimal preventive chemotherapy program. Several countries have proposed MDA approaches to eliminate STH infection [9–11], although the elimination goals for the short- and long-term may vary from country to country. Many mathematical models have been developed to optimize control strategies for the elimination of STH [9,12–14]. In Kenya, annual MDA for children has been implemented since 2012 to reduce STH infection below the prevalence of 1% [9]. The DeWorm3 project is focused on breaking the transmission of STH by using community-wide MDA (cMDA), conducted in study sites in Benin, India and Malawi, with the aim of reducing the prevalence of infection to less than 2% [10].

Mathematical models can be used to explore the dynamics of infectious diseases, including the spread of diseases, key epidemiological features of diseases, and changes in disease trends following an intervention [15]. The concept of STH transmission dynamic model has been developed to capture some changes of STH through number of mean worms [16]. The distribution pattern of worms in a host is characterized by aggregation; that is, a greater number of hosts harbor few or no worms, while a small number of hosts harbor a large number of worms. This distribution can be described using the negative binomial probability distribution. The degree of worm aggregation or “clumping” varies inversely with parameter *k*, defined as the aggregation parameter of worm burden distribution. The smaller the value of *k*, the greater the extent of aggregation. An age-structured model for STH has been developed to analyze worm distribution in different hosts to determine the results of age-targeted treatment control [12].

Mathematical models can be used to explore various control strategies for the elimination of STH [9,17–20]. Whether implementing MDA that targets only SAC could result in STH elimination remains controversial, as some models have suggested that MDA among SAC had little impact on reducing mean worm or prevalence [18]. By deploying a cMDA intervention, achieving STH elimination becomes more plausible [9,20]. Nevertheless, it will still be necessary to design and evaluate any potential elimination programs, including identification of the target population, the level of MDA coverage, and the frequency of MDA.

The aggregation of worms within hosts has been identified as having a considerable impact on STH surveillance and control programs [21]. For STH various species, both male and female worms must be present in the same host to produce eggs, reflecting in the degree of aggregation of worms in communities [22]. After many rounds of chemotherapy, the prevalence tended to fall, while the degree of aggregation increased with a low parameter *k* value [10,23]. The “clumping” increases the likelihood that parasites successfully locate members of the opposite sex within a human host. Hence, the breakpoint in infection is much lower when parasites are aggregated as compared to randomly distributed throughout the host population [23]. Another model demonstrated a strong negatively linear relationship between prevalence and worm aggregation [10], this was particularly the case when the prevalence was low.

In this study, we modeled the dynamics of mean worms in humans and the environment. We estimated the prevalence from the mean worm and used the model to investigate the impacts of various interventions, including the current practice and some potential adaptations, such as expanding target populations and/or varying frequency of testing/treatment. The target outcomes were a reduction in prevalence of STH according to the national (less than 5% prevalence of STH by 2026) and globally (less than 2% prevalence of STH by 2030) defined STH elimination goals. The model runs the 10-year projection between 2024–2033 to evaluate the potential for achieving the national target and global target.

## Methods

### Data collection: The Helminth control project

**Fig 1:**
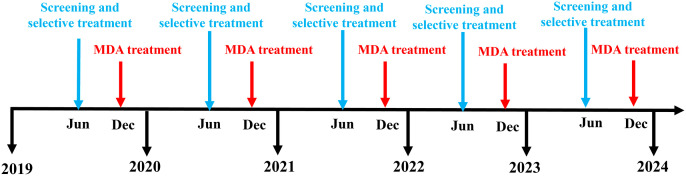
Current interventions in the helminth control project, conducted between 2019 and 2024.

We obtained datasets from the project under the responsibility of the Department of Disease Control, the Thai Ministry of Public Health, from 2002 to 2020 [8]. We will call this *“The helminth control project”* from now on. As part of the project, SAC has been screened annually for helminth infections since 2002. The prevalence of helminths has decreased considerably since the project started. Between 2015 and 2020, the prevalence showed fluctuations; the most common helminth infection was STH. The current control intervention involves using the KK test as a screening test to detect helminth infections. If an individual is infected, they receive selective treatment (i.e., a TnT). Six months later, MDA is carried out among SAC (Fig 1). This current intervention covered 914 schools in 55 provinces of Thailand and participants in the Phufa project, the selected areas were Bo Kluea and Chaloem Phra Kiat districts in Nan province. This intervention gives an estimated coverage of TnT and MDA of 80% and 95%, respectively, among SAC.

### Method assumptions

We developed a transmission dynamic model to describe the transmission of STH, incorporating three age classes of the population (PSAC, SAC, and adults), the dynamics of worm populations in the environment, and the various interventions. The model parameters were fitted to the yearly prevalence data from the helminth control project in Thailand. The model simulated the dynamics of STH between human hosts and the environment, taking into account worm biology (worm aggregation, mating probability, and density-dependent egg production) and other parameters such as the transmission rate and the contribution rate of infective stage for the different age groups. We used our model to explore some modifications of the current interventions, including TnT and MDA, and the embedding of the water, sanitation, and hygiene (WASH) principles, and to assess the impact on prevalence reduction and possibilities for achieving Thailand’s target (less than 5% prevalence of STH) and WHO’s target (less than 2% prevalence of STH) for STH elimination by 2030. The model structure was based on literature reviews of existing models with some modifications to fit the country context [9,12,19].

### STH transmission dynamic model conceptual framework

**Fig 2:**
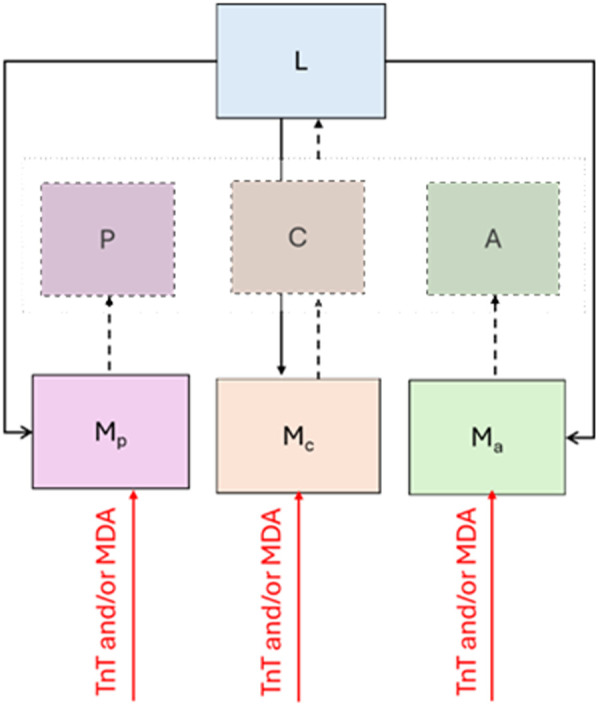
Schematic representation of the STH transmission dynamic model with interventions. The compartments represent both mean worm populations (M_p_, M_c_, M_a_) and estimated infections (P, C, A) from PSAC, SAC and adults respectively. The solid lines represent the flows from compartment to compartment in the ordinary differential equations, while the dotted lines represent the estimation of prevalence given the mean worm for each population.

The STH transmission dynamic model used in this study is shown in Fig 2. The three host groups were classified based on age groups as defined by WHO: children aged 0–4 years are classified as PSAC (P), children aged 5–14 years are classified as SAC (C), and individuals aged 15 years old and above are classified as adults (A). Worm populations were classified into two groups: mature worms in a human host (Mp,Mc, Ma) and free-living infective larvae in the environment (L).

The worm population in human hosts, or mean worm burden (Mp,Mc, Ma), is given by the balance of worm acquisition and the death rate of mature worms in hosts. An infected individual host contributes free-living infective larvae to the environment (L) at the rates (α_p_, α_c_, α_a_). Within the environment (L), the mean egg production rate (f) shown as Equation (1) is the mean egg production rate from a host population with mean worm burden M (requiring at least one mature male worm for a female worm to be able to produce fertile eggs) multiplied by the mating probability factor (ψ), shown as Equation (2). The term $e^{-\gamma }$ represents the density-dependency of egg production by worms in host [24] (Table 1).

**Table 1:** Description of model parameters and values.

Parameter description	Value	Source
Transmission rate in PSAC, SAC and adults (year^-1^), $\begin{array}{c} \beta_{p},\;\beta_{c},\;\beta_{a} \end{array}$	Estimated	Model fitting
Relative contributions of PSAC, SAC and adults to the environment, $\begin{array}{c} \alpha_{p},\alpha_{c},\alpha_{a} \end{array}$	Estimated	Model fitting
Aggregation parameters T1, T2, T3 and T4, k1, k2, k3, k4	Estimated	Model fitting
Average life span of worms (adults), 1/μw (days)	365	[9]
Average life span of worms (infective stage), 1/μL (days)	84	[9]
Strength of density-dependency of egg production, γ	0.0035	[24]
Proportion of PSAC in the population, np	0.06	[25]
Proportion of SAC in the population, nc	0.14	[25]
Proportion of adults in the population, na	0.8	[25]
Kato-Katz test sensitivity, ${\text{Kato}}_{\text{sen}}$ (%)	80	[26]
Drug efficacy (albendazole), h (%)	90	[27]
TnT coverage, ${TnT}_{cov}$ (%)	80 (for baseline)	See text
Frequency of TnT per year, ${timesperyear}_{TnT}$	1 or 2	Intervention design
Time interval between TnT rounds per year, ${time\_interv}_{TnT}$ (days)	vary	Intervention design
Time or duration of TnT, ${campaigndays}_{TnT}$ (days)	30	Intervention design
MDA coverage, gMDA, %	95 (for baseline)	See text
Frequency of MDA treatment per year, ${timesperyear}_{MDA}$	1 or 2	Intervention design
Time interval between MDA treatment rounds, ${time\_interv}_{MDA}$	vary	Intervention design
Time or duration of MDA, ${campaigndays}_{MDA}$ (days)	30	Intervention design


$$f\;=\;\left[\frac{M}{{\left[1+\;\frac{M}{k}\left(1-e^{-\gamma }\right)\right]}^{k+1}}\right]*\;\psi$$ (1)

$$\psi =1-\;{\left[\frac{1+\;\frac{M}{k}\left(1-e^{-\gamma }\right)}{1+\;\frac{M}{k}\left(2-e^{-\gamma }\right)}\right]}^{k+1}$$ (2)

The number of STH population can be described by the following ordinary differential equations (ODEs), shown as Equation (3)–(6).


$$\frac{dMp}{dt}=(\beta p*L)-(\mu w*Mp)-(TnTp\_p*Mp)-(MDAcampaign\_p*Mp)$$ (3)

$$\frac{dMc}{dt}=(\beta c*L)-(\mu w*Mc)-(TnTp\_c*Mc)-(MDAcampaign\_c*Mc)$$ (4)

$$\frac{dMa}{dt}=(\beta a*L)-(\mu w*Ma)-\;(TnTp\_a*Ma)-(MDAcampaign\_a*Ma)$$ (5)

$$\frac{dL}{dt}=\left(\left(fp*np*\;\alpha_{p}\right)+\left(fc*nc*\;\alpha_{c}\right)+\;\left(fa*na*\;\alpha_{a}\right)\right)\;-\;(\mu L+(\beta p\;*np)\;+(\beta c*nc)+(\beta a*na))*L$$ (6)

The relationship between parameter *k* and two epidemiological measures, the prevalence (P) and the intensity of infection (M) [17], is given by Equation (7).


$$P=1-\;{\left(1+\;\frac{M}{k}\right)}^{-k}\;$$ (7)

### STH transmission model with modified interventions

To explore the achievement of the target for STH elimination, we compared the impact between the current intervention, i.e., giving TnT in June followed by MDA in December each year (Fig 1) and the modified interventions where biannual MDA or biannual TnT was given based on the timeline of the current intervention. We also explored expanding the target populations including PSAC and adults with 50% and 80% coverage of these modified interventions (Table 2).

**Table 2:** Summary of designed intervention on difference targeting population and coverage.

Strategy	Frequency				Coverage (%)					
	TnT		MDA		TnT			MDA		
	1/year	2/year	1/year	2/year	PSAC	SAC	Adults	PSAC	SAC	Adults
**Current intervention (baseline)**										
1. Expanded target groups	✓	☓	✓	☓	☓	80	☓	☓	95	☓
1.1 Targeting SAC and adults	✓	☓	✓	☓	☓	80	50 or 80	☓	95	50 or 80
1.2 Targeting SAC and PSAC	✓	☓	✓	☓	50 or 80	80	☓	50 or 80	95	☓
1.3 Targeting PSAC, SAC, and adults	✓	☓	✓	☓	50 or 80	80	50 or 80	50 or 80	95	50 or 80
**2. Biannual MDA**										
2.1 Targeting only SAC	☓	☓	☓	✓	☓	☓	☓	☓	95	☓
2.2 Targeting SAC and adults	☓	☓	☓	✓	☓	☓	☓	☓	95	50 or 80
2.3 Targeting SAC and PSAC	☓	☓	☓	✓	☓	☓	☓	50 or 80	95	☓
2.4 Targeting PSAC, SAC, and adults	☓	☓	☓	✓	☓	☓	☓	50 or 80	95	50 or 80
**3. Biannual TnT**										
3.1 Targeting only SAC	☓	✓	☓	☓	☓	80	☓	☓	☓	☓
3.2 Targeting SAC and adults	☓	✓	☓	☓	☓	80	50 or 80	☓	☓	☓
3.3 Targeting SAC and PSAC	☓	✓	☓	☓	50 or 80	80	☓	☓	☓	☓
3.4 Targeting PSAC, SAC, and adults	☓	✓	☓	☓	50 or 80	80	50 or 80	☓	☓	☓

### Model validation

We used R software version 4.2.1 to run and analyze the model output, and the *deSolve* package to solve the ordinary differential equations (ODEs) [28]. The initial parameter values were calculated from population and disease burden data. Model fitting was carried out using the Markov chain Monte Carlo (MCMC) simulation, implemented with the *BayesianTools* R package [29]. The model was run using time unit of a day and fitted to the prevalence of helminth infections in Thailand from 2002 to 2020. Ten parameters were estimated, and the median values and credible intervals (95% CrI) were reported and used as the baseline scenario for the current intervention for the 10-year prevalence projection from 2024 to 2033 (see the S1 Text). The model was further used to explore the modifications to TnT and MDA and predict helminth infections and the helminth population in the environment. The model predictions are reported as prevalence data for each target group (PSAC, SAC, and adults) and the mean worm number in humans and the environment.

## Results

### Overview of the helminth control project performance

**Fig 3:**
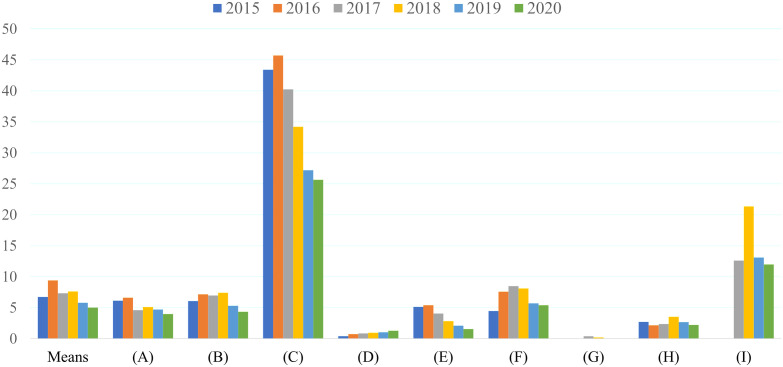
The overall prevalence of any helminth infections between 2002 and 2020 based on the school types. (A) Border patrol police schools, (B) Schools under the Office of the Basic Education Commission, (C) Community learning centers of the Office of the Non-formal Education Commission, (D) Monastic schools under the National Office of Buddhism, (E) Private Islamic schools under the Office of the Private Education Commission, (F) Schools under the local government and child development centers, (G) Schools under the Bangkok Metropolitan Administration (BMA), and (H) Rajaprajanugroh schools.

Since 2002, SACs were screened for helminth infections as part of the project. The prevalence of helminths has decreased considerably since the helminth control project started, from 26.78% in 2002 to 5.01% in 2020. In 2015–2020, there were some variations in STH rates among different areas or different school types including (A) Border patrol police schools, (B) Schools under the Office of the Basic Education Commission, (C) Community learning centers of the Office of the Non-formal Education Commission, (D) Monastic schools under the National Office of Buddhism, (E) Private Islamic schools under the Office of the Private Education Commission, (F) Schools under the local government and child development centers, (G) Schools under the Bangkok Metropolitan Administration (BMA), and (H) Rajaprajanugroh schools. Some school types demonstrated a considerably higher prevalence than the average, while others exhibit a markedly lower prevalence. SAC under the community learning centers (C) had high STH infection rates (Fig 3). Most of these schools are situated along the western border of Thailand and Myanmar, an area known for its low-socio-economic status and mixed population of Thai and Karen refugees. The high prevalence of STH infections among SAC in these schools is attributed to poor sanitation, inadequate personal hygiene, and limited access to clean water in the community. In contrast, other schools have a lower prevalence of STH infections due to better access to sanitation facilities and clean water for both the children and the community. The most common STH is caused by *A*. *lumbricoides* (see the S2 Text).

### Parameter estimation through model fitting

**Fig 4:**
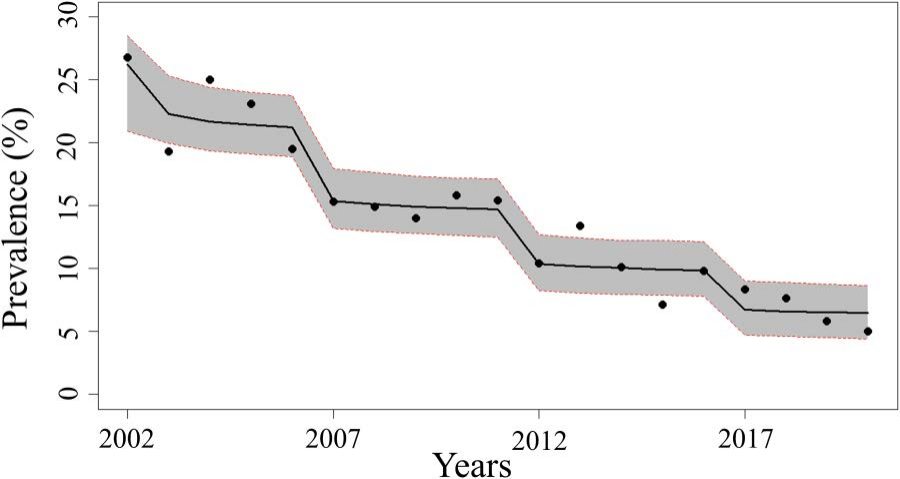
Comparing the observed and model estimates of prevalences of STH in Thailand between 2002 and 2020. The black dots indicate observed prevalence data, and the black line indicates model estimate prevalences. The red dotted line indicates the 95% credible intervals (CrI) of model estimates.

The model parameters estimated from the best-fit model using the Bayesian method are presented in Table 3. The model fitting the STH prevalence data over time is shown in Fig 4 The transmission rates in each population were estimated to be 1.972 (95% CrI: 0.872–4.855), 1.536 (95% CrI:0.557–3.438), and 0.662 (95% CrI: 0.414–1.390) in PSAC, SAC, and adults, respectively. The contributions of host infections to the free-living infective larval stages in the environment were estimated to be 2.768 (95% CrI: 1.215–6.385), 2.726 (95% CrI: 1.252–5.680), and 2.675 (95% CrI: 1.718–4.509) in PSAC, SAC, and adults, respectively. The aggregation parameters (*k*) were assumed to vary over time and were calibrated in four time-intervals, i.e., T1 was between 2002 and 2006, T2 was between 2007 and 2011, T3 was between 2012 and 2016, and T4 was between 2017 and 2020.

**Table 3:** Results of parameters estimated for the STH transmission dynamic model.

Parameter	Symbol	Median value (95% CrI)
Transmission rate in PSAC (year^-1^)	$\beta_{p}$	1.972 (0.872–4.855)
Transmission rate in SAC (year^-1^)	$\beta_{c}$	1.536 (0.557–3.438)
Transmission rate in adults (year^-1^)	$\beta_{a}$	0.662 (0.414–1.390)
Relative contributions of PSAC to the environment (year^-1^)	$\alpha_{p}$	2.768 (1.215–6.385)
Relative contributions of SAC to the environment (year^-1^)	$\alpha_{c}$	2.726 (1.252–5.680)
Relative contributions of adults to the environment (year^-1^)	$\alpha_{a}$	2.675 (1.718–4.509)
Aggregation parameter T1	$k_{\text{1}}$	0.042 (0.041–0.045)
Aggregation parameter T2	$k_{\text{2}}$	0.028 (0.027–0.030)
Aggregation parameter T3	$k_{\text{3}}$	0.018 (0.017–0.030)
Aggregation parameter T4	$k_{\text{4}}$	0.011 (0.011–0.012)

### Impact of interventions

#### Expanding the current intervention to other target groups.

**Fig 5:**
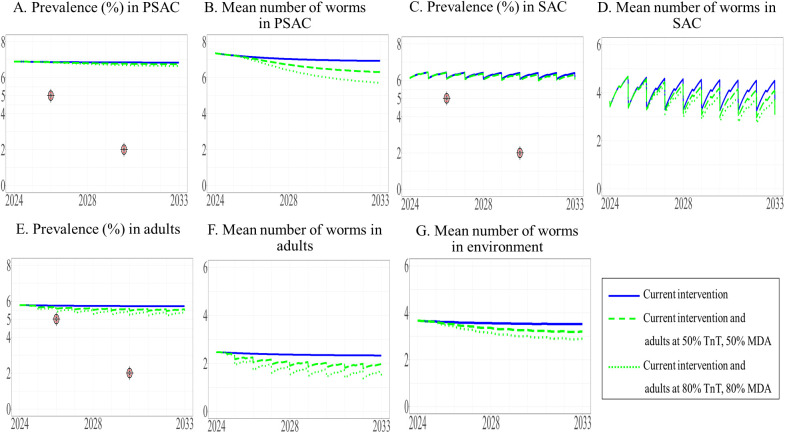
Model prediction of the prevalence of STH and mean worm number between 2024 and 2033 under the current intervention with its expansion to adults. The prevalence of STH and mean number of worms in PSAC (A and B), SAC (C and D), and adults (E and F) are shown, with the mean number of worms in the environment shown in G.

**Fig 6:**
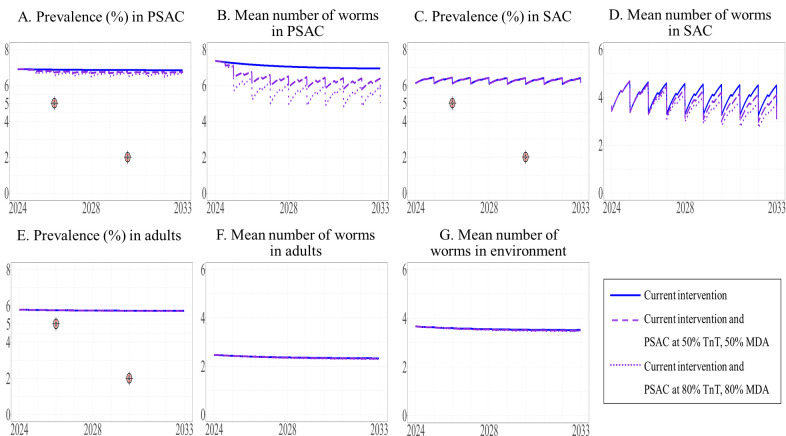
Model prediction of the prevalence of STH and mean worm number between 2024 and 2033 under the current intervention with its expansion to PSAC. The prevalence of STH and mean number of worms in PSAC (A and B), SAC (C and D), and adults (E and F) are shown, with the mean number of worms in the environment shown in G.

**Fig 7:**
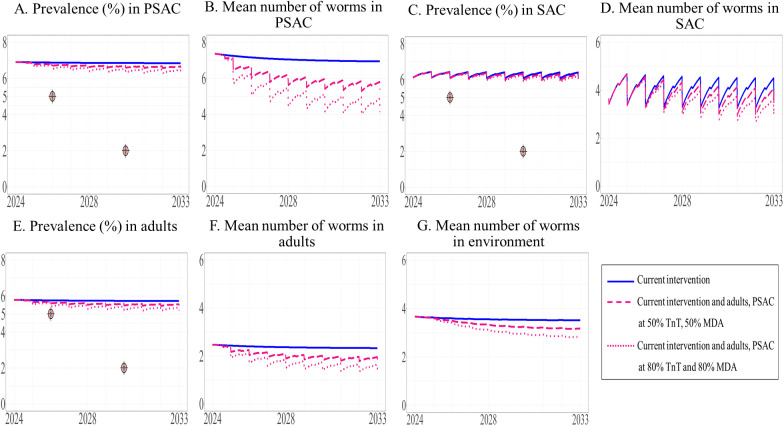
Model prediction of the prevalence of STH and mean worm number between 2024 and 2033 under the current intervention with its expansion to both adults and PSAC. The prevalence of STH and mean number of worms in PSAC (A and B), SAC (C and D), and adults (E and F) are shown, with the mean number of worms in the environment shown in G.

We examined the effects of expanding the current interventions to other target groups, including adults (Fig 5), PSAC (Fig 6), and both of these groups (Fig 7), at the same time allowing two levels of coverage of these interventions, i.e., 50% and 80%. The model projected prevalence and mean worm burden over time. Over the 10-year period, the prevalence of STH in SAC remained stable (estimated to be 5%) under the current intervention and did not decrease to the defined elimination target level of less than 5%. Similarly, the mean worm burden in SAC was relatively stable (estimated to be between 3.222 and 3.410) (Table 4).

**Table 4:** Time series prevalence of STH and worm number estimate data under the current intervention targeting SAC only.

Year	Prevalence (%)			Worm number in population			
	PSAC	SAC	Adults	PSAC	SAC	Adults	Environment
2024	6.91	6.19	5.79	6.566	3.410	2.192	3.094
2025	6.89	6.17	5.77	6.455	3.358	2.155	3.057
2026	6.88	6.16	5.76	6.373	3.320	2.128	3.030
2027	6.87	6.15	5.75	6.313	3.292	2.107	3.009
2028	6.86	6.15	5.74	6.267	3.270	2.092	2.994
2029	6.86	6.14	5.74	6.233	3.254	2.081	2.982
2030	6.85	6.14	5.73	6.208	3.242	2.072	2.973
2031	6.85	6.14	5.73	6.189	3.233	2.066	2.967
2032	6.85	6.14	5.73	6.175	3.227	2.061	2.962
2033	6.85	6.13	5.73	6.164	3.222	2.058	2.958

Our results indicated that the overall prevalence of STH in the three populations under the modified intervention expanded to adults or PSAC did not differ from the baseline prevalence. In addition, even with the expansion to all three populations, the prevalence of STH did not decrease to the elimination level (see the S3A and S3B Table).

#### Biannual MDA intervention.

**Fig 8:**
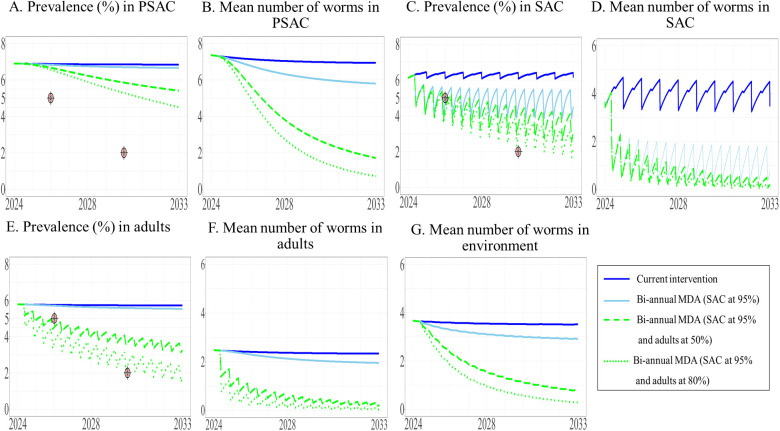
The model’s predictions of prevalence of STH and mean worm number between 2024 to 2033 under the current intervention compared with biannual MDA expanded to adults. The prevalence of STH and mean number of worms in PSAC (A and B), SAC (C and D), and adults (E and F) are shown, with the mean number of worms in the environment shown in G.

**Fig 9:**
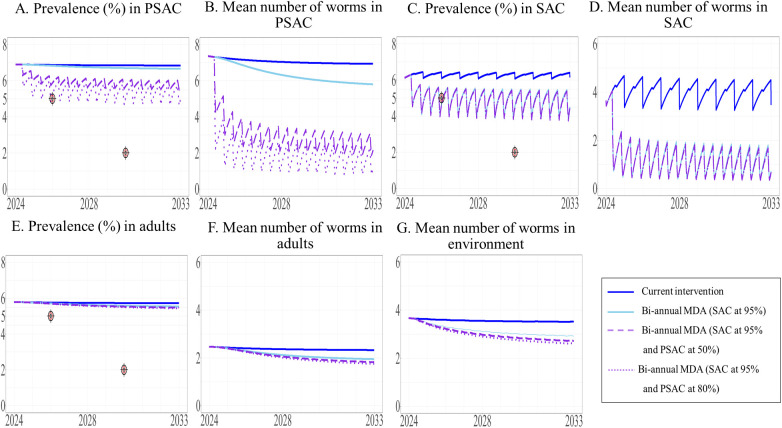
The model’s prediction of prevalence of STH and mean worm number between 2024 to 2033 under the current intervention compared with biannual MDA expanded to PSAC. The prevalence of STH and mean number of worms in PSAC (A and B), SAC (C and D), and adults (E and F) are shown, with the mean number of worms in the environment shown in G.

**Fig 10:**
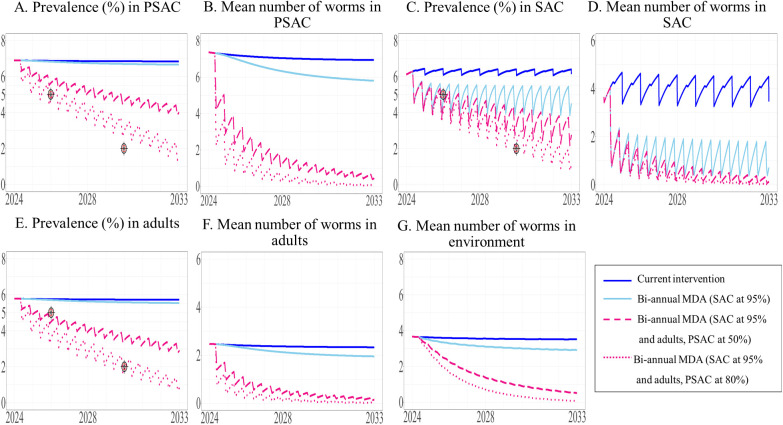
The model’s prediction of prevalence of STH and mean worm number between 2024 to 2033 under the current intervention compared with biannual MDA expanded to both adults and PSAC. The prevalence of STH and mean number of worms in PSAC (A and B), SAC (C and D), and adults (E and F) are shown, with the mean number of worms in the environment shown in G.

We modeled the effect of modifying the current intervention by substituting TnT with the implementation of biannual MDA in different target populations. The model predicted that all biannual MDA treatment scenarios would reduce the prevalence of STH in SAC to less than 5% by 2026. Our results indicated that expanding the intervention to include adults would be more effective than targeting PSAC, as illustrated in Fig 8. Implementing biannual MDA for SAC at 95% coverage and expanding this to adults at 50% and 80% coverage was predicted to result in a reduced prevalence of STH in SAC to less than 5% by 2026, with 4.64% and 4.56%, respectively (Fig 8). These two above scenarios could further reduce the prevalence of STH in SAC to 3.15% and 2.18% by year 10. In addition, biannual MDA targeting only SAC and expanding this to PSAC at 50% and 80% coverage decreased prevalence to 4.72%, and 4.71%, respectively, by 2026 (Fig 9). Between 2024 and 2033, the prevalence of STH in SAC was predicted to consistently remain at 4%. Implementation of biannual MDA targeting all three populations at 95% coverage for SAC and 50% and 80% coverage for adults and PSAC resulted in a reduction in overall prevalence of STH to 4.63% and 4.55% by 2026 (see the S3C Table). The model predicted that only biannual MDA targeted at adults and PSAC at 80% coverage would be able to achieve WHO’s goal (prevalence less than 2%) just after 2030 where the target was set for (Fig 10). In summary, all biannual MDA strategies would be able to achieve Thailand’s national elimination target within one year.

We used the model to predict both the mean worm burden and the prevalence of STH. We observed a correlation between prevalence and mean worm burden in each population, (see the S3C and S3D Table). When the prevalence of STH ranged from 5% to 2%, the mean worm burden ranged from 0.882 to 0.070 in SAC, from 1.068 to 0.079 in adults, and from 0.981 to 0.062 in PSAC. Additionally, when the prevalence was less than 2%, the mean worm burden was less than 0.052 in SAC, 0.055 in adults, and 0.037 in PSAC. Under biannual MDA, the number of helminths in the environment has decreased in correlation with mean worm number in host

#### Biannual TnT intervention.

**Fig 11:**
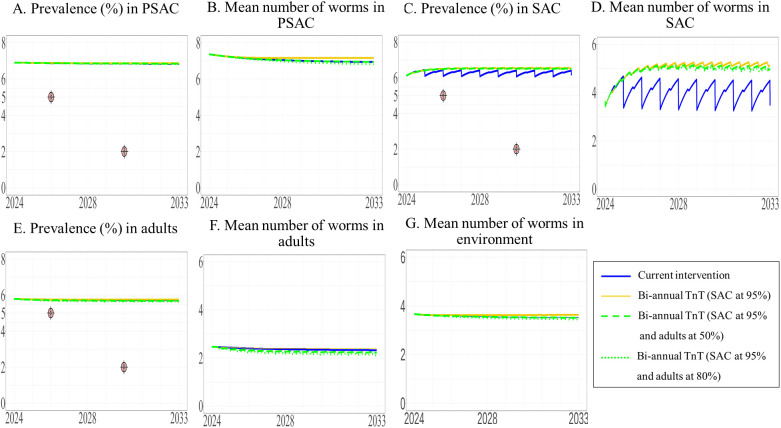
The model’s prediction of prevalence of STH and mean worm number between 2024 to 2033 under the current intervention compared with biannual TnT expanded to adults. The prevalence of STH and mean number of worms in PSAC (A and B), SAC (C and D), and adults (E and F) are shown, with the mean number of worms in the environment shown in G.

**Fig 12:**
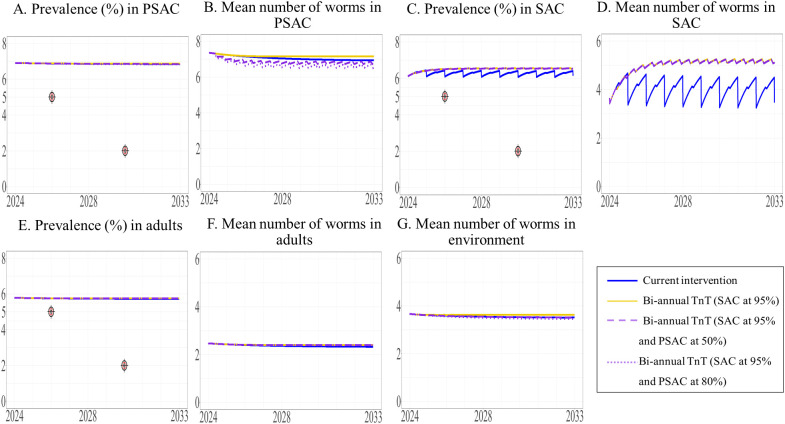
The model’s prediction of prevalence of STH and mean worm number between 2024 to 2033 under the current intervention compared with biannual TnT expanded to PSAC. The prevalence of STH and mean number of worms in PSAC (A and B), SAC (C and D), and adults (E and F) are shown, with the mean number of worms in the environment shown in G.

**Fig 13:**
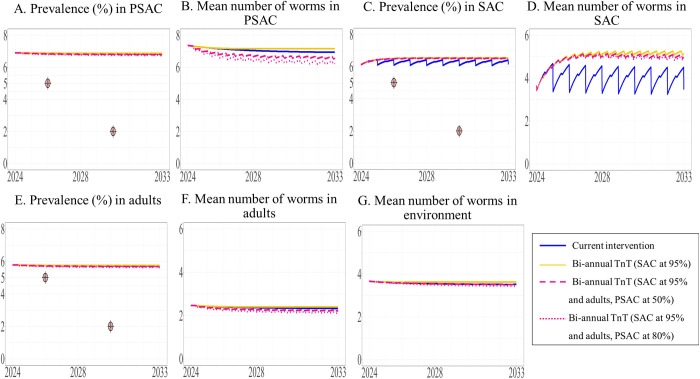
The model’s prediction of prevalence of STH and mean worm number between 2024 to 2033 under the current intervention compared with biannual TnT expanded to both adults and PSAC. The prevalence of STH and mean number of worms in PSAC (A and B), SAC (C and D), and adults (E and F) are shown, with the mean number of worms in the environment shown in G.

We modeled the effect of biannual TnT on different target populations and with different coverage levels. We found that expanding the intervention to include adults and/or PSAC would not achieve the elimination target level of a prevalence of less than 5%, (see Figs 11, 12, and 13 and the S3E Table). Additionally, the prevalence of STH and mean worm burden under the biannual TnT did not differ significantly from those of the current intervention S3E and S3F Table).

## Discussion

This study highlights important parameters for STH transmission and the impact of the school-based control program, consisting of annual TnT and MDA interventions, for STH infections among children in rural areas of Thailand. The STH transmission dynamic model we used was developed based on STH life cycles and transmission dynamics. The transmission dynamics of the various species responsible for STH are identical due to the direct life cycle they share in common. The time delay from infection to establishment in the human intestine as an adult worm is similar in all four species and generally much shorter than the life span of the worm in the host and so contributes little to differentiating the species. Differences in the mechanisms of worm establishment are not significant in the context of population-level descriptions of changing parasite burdens [30]. In the present study, we selected *A*. *lumbricoides* as representative of STH for our transmission model because in Thailand *A*. *lumbricoides* infection predominantly affects SAC. We observed a decreasing value as the prevalence of STH infections decreased. We used our STH transmission dynamic model to evaluate the impact of the current control program and modified interventions, including biannual MDA and biannual TnT. We demonstrated that the coverage level and target population are critical factors for the effectiveness of these interventions. Additionally, our study showed that population characteristics are essential considerations when designing interventions to control STH.

Soil-transmitted helminthiases are common among children in rural areas of Thailand. However, the prevalence of STH has been decreasing following multiple rounds of the school-based control program. A small number of infected individuals contribute fertilized eggs to the environment, which results in new infections. Repeated rounds of annual treatment have reduced overall levels of infection in the community due to a decline in the release of fertilized eggs. The change in prevalence of STH exhibited a strong linear correlation with the degree of parasite aggregation, as measured inversely by the negative binomial parameter *k*. Lower prevalence is associated with greater aggregation. As prevalence decreases, worms appear to become increasingly concentrated in just a few individuals [30]; this indicates that populations with lower prevalences are less affected by treatment compared with those with higher prevalences [10].

The Thai Ministry of Health has set a roadmap to eliminate STH by 2026, defined as a prevalence of STH of less than 5%. The current control program has focused on SAC, using a combination of interventions, including TnT and MDA. This control program was initially effective but eventually reached a plateau and is now unlikely to achieve the target prevalence level. School-based MDA strategies have been employed in many countries but have frequently been unable to eliminate STH [10,11,31]. The most recent WHO guidelines recommend that at-risk populations should receive MDA, while a mathematical model has shown the effectiveness of community-based deworming [7]. The coverage, frequency, target population, and the performance of diagnostic tests are all key aspects to be considered for modified interventions [13]. We used our model to predict STH transmission dynamics and prevalence under modified interventions to be carried out for 10 years between 2024 and 2033. The model demonstrated that biannual MDA, with 80% coverage in all three populations could reduce prevalence to the elimination levels, i.e., below 5% and 2% prevalence. Conversely, TnT, whether annual or biannual, were not predicted to be effective in reducing STH. This is because when the prevalence is low, fewer individuals are infected, making it difficult to reach them for testing due to limitations in coverage or test performance. The worm burden is reduced due to repeated treatments but has yet to be driven below the breakpoint. Transmission will be continued in the community [13]. We next sought to predict the impact of expanding modified interventions to include PSAC and adults.

To advance toward the elimination phase for STH, control strategies must emphasize repeated rounds of MDA with high coverage, defined as individuals receiving the treatment. However, this is also dependent on compliance, i.e., each individual who receives treatment actually taking the treatment [32]. Individuals who exhibit non-compliance with an MDA initiative have the potential to be predisposed to STH [33]. Systematic non-compliance refers to the proportion of a population who remain untreated following consecutive MDA treatment rounds [34]. In Thailand, this non-compliance may manifest among SAC who do not receive treatment and other population groups who never receive drug treatment. Our model projected that implementing biannual MDA targeting only SAC at 95% coverage or expanding to adults and PSAC at 50% or 80% coverage would have the potential to achieve Thailand’s elimination target. All biannual MDA strategies were shown to drive the prevalence of STH in SAC to approximately 4% by the first year of implementation. We found that expanding the intervention to adults would be more effective than expanding it to PSAC. Moreover, biannual MDA strategy that was adults and PSAC at 80% coverage reduced prevalence to less than 2%, WHO’s target elimination level just a year after the target elimination was set. The reason for these different impacts of the interventions on the respective target populations is because of the high proportion of adults (80%) in the population compared with the low proportion of PSAC (6%). In Myanmar, it was shown that, together, adults and SAC harbor approximately 94% of overall STH infections, with 80% to 90% of the total eggs per gram (EPG) count was contributed by adults and SAC [35]. Expanding MDA treatment to other groups, particularly those with either a high intensity of infection or high levels of non-compliance could have the potential to interrupt STH transmission. Implementation of deworming in school is facing challenges, due to a considerable proportion of children in many low-and middle-income countries not attending school [36]. In Bangladesh, limitations of a biannual school-based MDA intervention were reported to be due to the target population (children who were not attending school), difficulties in achieving MDA coverage (drug distribution policy, accessibility to schools, poor record-keeping, and insufficient training of drug distributors), and inadequate information about population dynamics; additionally, misunderstandings about the possible side effects of the drugs resulted in non-compliance with MDA [37]. Community learning centers were one type of school in the helminth control project, which were identified with a high prevalence of infection. Managing or implementing mass drug administration (MDA) programs in this setting was challenging compared to the school system, mainly due to a lack of teachers – students’ cooperation and communication.

A further issue to consider is that MDA can only temporarily reduce the transmission of STH and cannot prevent reinfection. Furthermore, the repeated implementation of MDA may increase the risk of anthelmintics resistance [38]. MDA is highly beneficial in settings where the transmission risk is low, and the health system is strong. On the other hand, settings where there is a high transmission risk may require high-coverage, high-frequency, and broader community-based treatment, with additional health education and environmental sanitation improvements [38]. However, there are various barriers to adults participating in cMDA programs, such as adults’ mistrust of cMDA programs, their fear of side effects of deworming drugs, their perceived low risk of acquiring STH, and their absence during drug distribution drives [39]. The combination of MDA and WASH intervention is a potential alternative control program. WASH interventions are anticipated to help interrupt STH transmission, making it possible to eventually cease MDA activities. A mathematical model has been used to demonstrate the combined effect of MDA and WASH interventions, including health education and sanitation control [14]. Control programs that involve combined interventions were shown to be more effective than any single intervention. The transmission rate was identified as the most positively sensitive parameter. WASH interventions can directly decrease the transmission rate and break the cycle of transmission by increasing awareness of STH and promoting personal hygiene, along with providing clean water and installing public toilets. This combination can reduce environmental contamination and lead to more sustainable control and eventual elimination of STH.

For the test and treat (TnT) in the school-based control strategy in Thailand, the KK method is used to determine individuals’ infection status; selective treatment is then implemented for children who test positive. This method identifies a helminth infection by detecting helminth eggs, with the worms’ reproductive properties and density being important factors in egg production. This can impact the sensitivity of the KK method, especially in areas with a low prevalence of STH. Soil-transmitted helminths are dioecious, meaning that female worms require male worms to fertilize them and produce eggs. It is these eggs that are detected by the KK method. In low-prevalence settings, STH infection can be underestimated using the KK method, while some areas with the highest initial prevalence can remain hotspots for reinfection even after a treatment intervention [40]. Notably, the majority of helminth infections are of light intensity [41]. Poor detection of STH infection is a common issue in many resource-limited countries, such as Thailand, where the KK method remains the standard diagnostic test used throughout the country. Our previous work has shown that in settings with a low prevalence of STH, molecular techniques have greater sensitivity than the KK method. However, neither KK nor molecular methods performed well when the prevalence of infection was very low (<2%) [26]. Therefore, enhancing the sensitivity of diagnostic tests, such as by using a combination of two or more diagnostic tests, or the development of new methods for surveillance at the pre-elimination phase, should be considered.

Our study had some limitations both in terms of the model and the data. Generally, many STH published models would either deal with human population level alone [14,42] or macro-parasite population [17–20]. We attempted to hybrid the two levels with the focus on projecting the impacts of screen and treatment strategies. Our model structure could be too simplified for capturing the complexity of STH dynamics in great details. We modelled the dynamics of worms and used them to estimate the prevalences of infections in each population. We did not account for the dynamics of male and female egg interactions where this would be much more accurate process of worm production. In terms of data limitation, although we developed an STH transmission dynamic model to predict the impact of interventions on STH prevalence and mean worm burden, we were limited by the mean worm data due to the lack of quantitative counts of worms or eggs (only the positivity of a sample) in the routine sample process. We parameterized our model to the dominant STH species, *A. lumbricoides* although the development of mathematical models for other common species such as *T. trichiura* and hookworms would be useful for understanding transmission dynamics in areas where these two helminth species are also prevalent.

WHO has established targets for 2030 that are focused on achieving the elimination of STH transmission instead of morbidity control. In Sri Lanka, the national prevalence of STH is less than 1% in children but in some areas the prevalence is much higher than this. Therefore, the routine mass drug strategy might be discontinued at national level and should be targeted to selected areas that remain at high risk of STH [11]. In Thailand, the current control program is implemented repeatedly, targeting children in rural areas and based on school types. Hotspots of infection have been identified in some schools located in rural areas with a low level of sanitation. The planning of future control program strategies should focus on additional benefits, including cMDA and detection of infection hotspots. Expanding the target population to a community-wide approach has been suggested as an alternative option to accelerate STH elimination [20].

Future studies should consider tailoring the STH elimination champagne to specific geographical areas and prevalence, including selection of appropriate diagnostic testing. Additionally, anthelmintic resistance should be monitored [43], especially in areas where MDA has been implemented for a long period. Cost data of STH infections and interventions should be explored when some economic studies with cost-effectiveness of deworming programs will be important for the next steps in policy making.

## Conclusion

We developed an STH transmission dynamic model, which predicted that a biannual MDA would be more effective than the current STH control program in Thailand. Implementing the biannual MDA scenario was predicted to reduce the prevalence of STH to less than 5% in one year. Scaling up biannual MDA treatment to community-wide treatment would help in accelerating progress toward the STH elimination goal. Our results provide crucial data for public health policymakers when planning STH control programs aimed at eliminating STH within the national and global timelines.

## Supporting information

S1 Text Model fittin. (S1_Text.DOCX)

S2 Text The controlling helminth infection in children and youth in remote areas project. (S2_Text.DOCX)

S3 Table Prevalence and mean worm number. (S3_Table.DOCX)

S4 Text R code. (S4_Text.DOCX)
