# Supplementary material for: Mathematical modeling of control strategies for the elimination of soil-transmitted helminthiases in Thailand
Source: PLoS Negl Trop Dis. 2025 Aug 22;19(8):e0013435. doi: 10.1371/journal.pntd.0013435 (PMC12373168; doi:10.1371/journal.pntd.0013435)
Supplement: S1 Text — (S1_Text.DOCX) [file pntd.0013435.s001.docx]

**Mathematical modeling of control strategies for the elimination of soil-transmitted helminthiases in Thailand**

**Supporting information S1 Text**

*The Bayesian framework*

Bayesian analysis is used to estimate and update the posterior probability distribution of parameters of interest, given the observed data and prior information.

Bayes’ theorem states:

$$P\left( A | B \right) =\frac{P\left( B | A \right) * P\left( A \right)}{P\left( B \right)}$$

$P\left( A | B \right)$ is a conditional probability: the probability of event A occurring given B that is true. It is also called the posterior probability of A given B.

$P\left( B | A \right)$ is a conditional probability: the probability of event B occurring given A that is true. It is also called the likelihood of A given B because $P\left( B | A \right)$ = $L \left( A | B \right)$.

$P\left( A \right)$ and $P\left( B \right)$ are the probabilities of observing A and B, respectively, without any given conditions; they are known as the prior probability and marginal probability.

*Prior distribution*

The normal distribution was chosen to be the prior distribution for three beta parameters and three alpha parameters values in preschool-age children (PSAC), school-age children (SAC), and adults, with mean = 1 and standard variance = 0.5. Uniform distribution was chosen for four *k* parameter values at specific times, with a minimum value = 0.001 and a maximum value = 0.1. We expected that three beta parameters and three alpha parameters values are likely to fall within falls a specific range, allowing the mean to be set to this central value, while we have a little information value of four k parameters, so the uniform distribution allows us to express that all values within the specified range are equally likely.

*Likelihood function*

We defined the likelihood as the product of the likelihood term for each data point. The data were obtained from the observed prevalence of STH. These data points were linked to the model’s predictions using a Poisson distribution. The log-likelihood, used as the target in the Markov chain Monte Carlo (MCMC) algorithm, is:

Likelihood function of the model

$$\log L \left( \theta\right)= \sum_{i=1}^{n} (\text{ }X_{i}\log\lambda_{i}- \lambda_{i}- \log X_{i}!)$$

Where $X_{i}$ represents the observed positive cases in the helminth control project at time $i$, and $\lambda_{i}$ represents the expected number of STH in SAC predicted by the model at the same time.

*Posterior estimation*

We used a Differential Evolution Markov chain Monte Carlo (MCMCzs, or DE-MCzs) method to estimate the posterior distributions. We considered Markov chain methods of sampling that were proposed by Ter Braak and Vrugt et al. (2008) [1], which have previously been used for numerical problems, and implemented them using the Bayesian Tools R package. Differential Evolution Markov chain (DE-MC) is an adaptive MCMC algorithm, in which multiple chains are run in parallel and presented. The MCMC settings were configured to provide a robust estimation of the posterior distribution for each parameter, ensuring that the results are reliable and reflective of the underlying data. Two chains were run in parallel on three cores, 150000 iterations with the burn-in period of 15,000 time-steps were processed. The diagnostic checks including trace plots and calculated Gelman-Rubin statistics (PSRF) were monitored to ensure proper mixing of the chains and convergence.


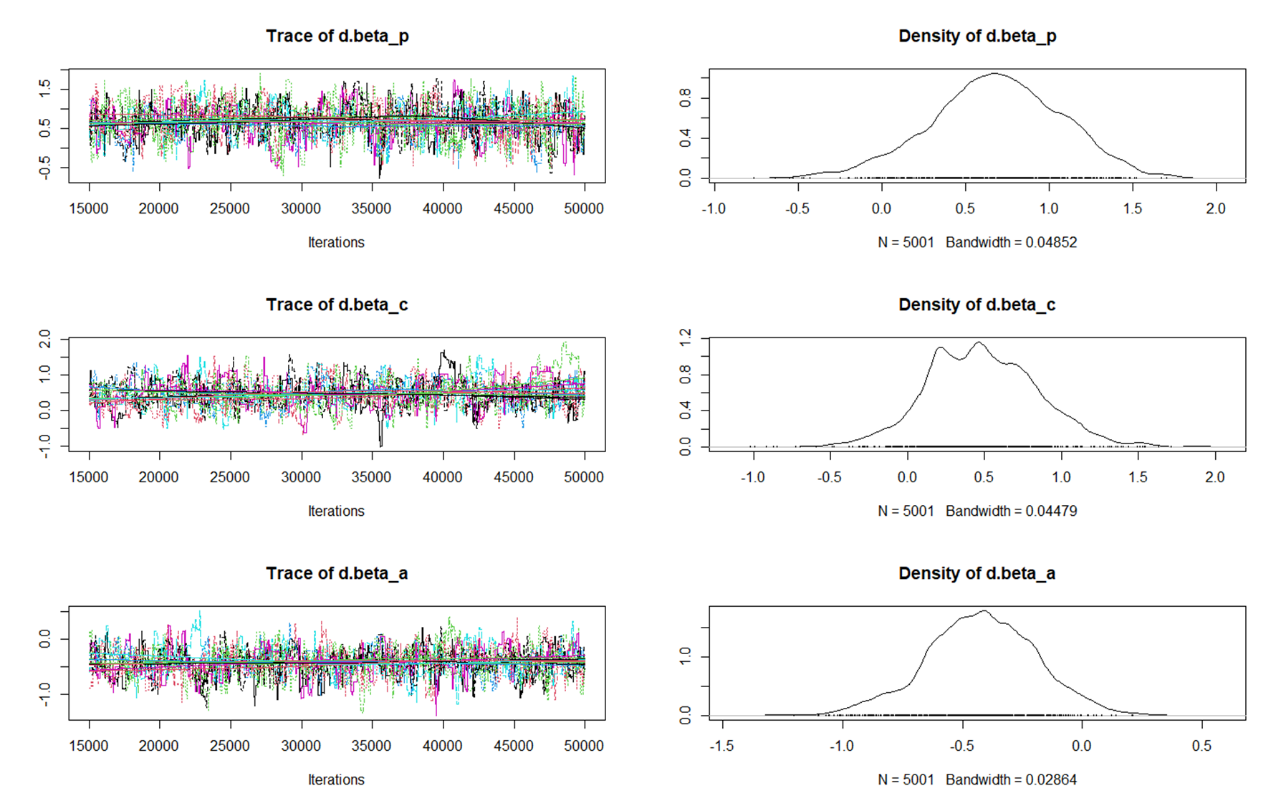


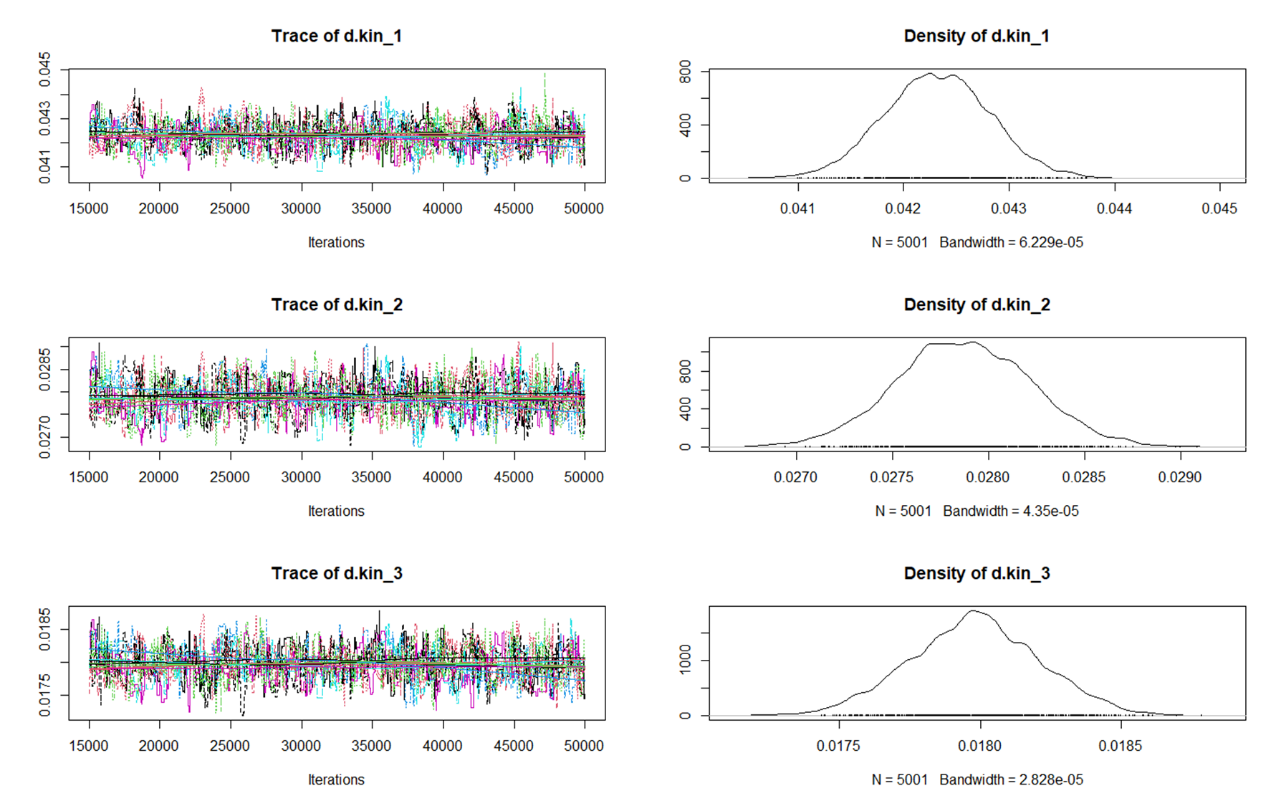

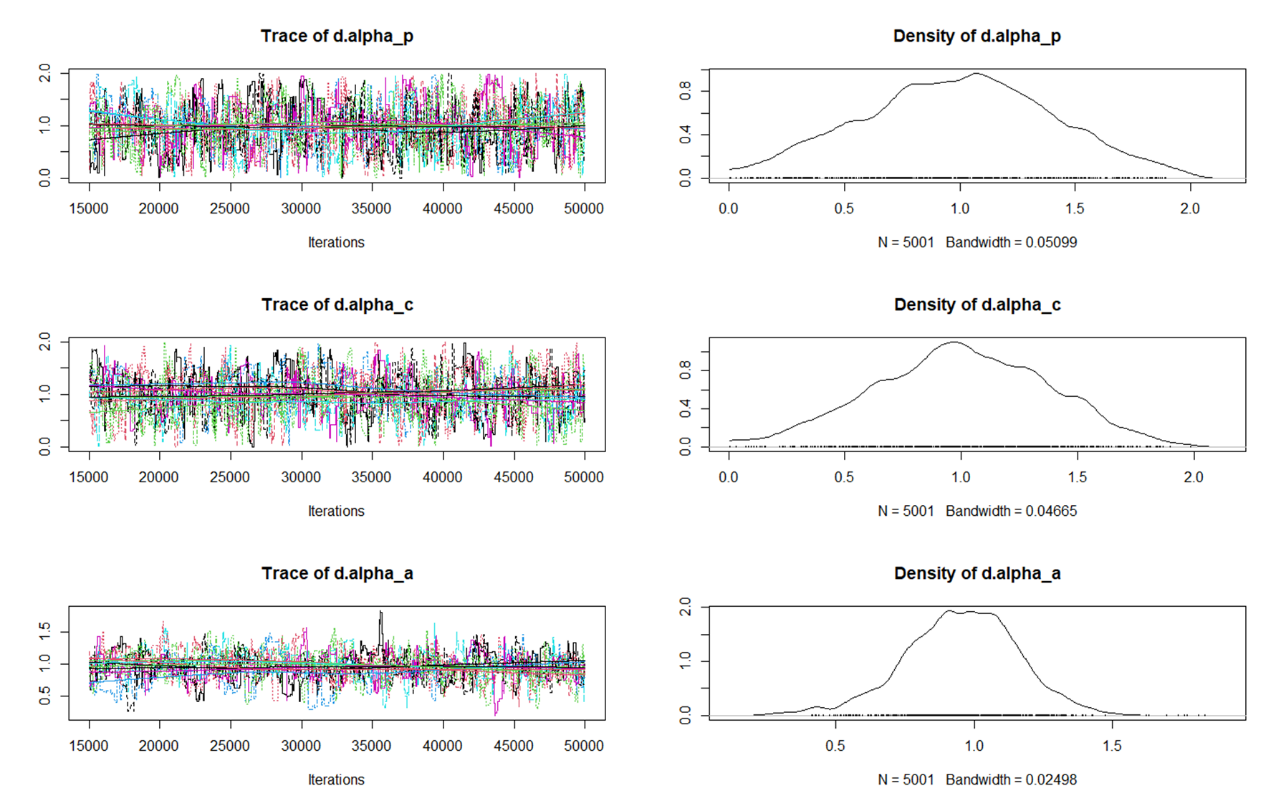


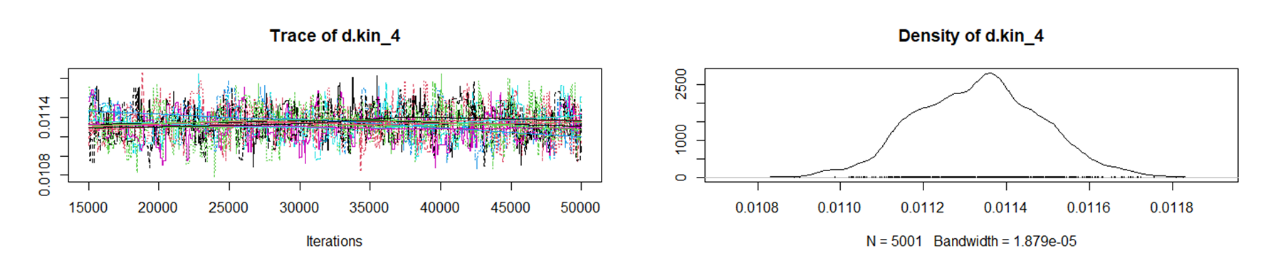


**Figure A. Posterior distributions from the STH transmission dynamic model. Each row corresponds to a separate parameter, the left-hand column contains traces with six color chains (dashed lines: actual traces, solid lines: trends) and the right-hand column contains the posterior distributions, corresponding to each parameter.**

References

1. Ter Braak CJ, Vrugt, J.A. Differential evolution Markov chain with snooker updater and fewer chains. Stat Comput 2008;18:435–46.
