# Supplementary material for: Mathematical modeling of control strategies for the elimination of soil-transmitted helminthiases in Thailand
Source: PLoS Negl Trop Dis. 2025 Aug 22;19(8):e0013435. doi: 10.1371/journal.pntd.0013435 (PMC12373168; doi:10.1371/journal.pntd.0013435)
Supplement: S2 Text — (S2_Text.DOCX) [file pntd.0013435.s002.docx]

**Mathematical modeling of control strategies for the elimination of soil-transmitted helminthiases in Thailand**

**Supporting information S2 Text**

*Project: Controlling helminth infection in children and youth in remote areas*

Soil-transmitted helminthiases (STH) predominantly occur in rural areas and mostly in children. In Thailand, a national survey of helminthiases in children was started in 2002, namely controlling helminth infection in children and youth in remote areas project, overseen by the Department of Disease Control, part of the Thai Ministry of Public Health [1-3]. In 2002, the baseline overall prevalence of any STH was 26.78% (Fig A). The Phufa project had a considerable impact, with STH prevalence decreasing to less than 10% in 2015.


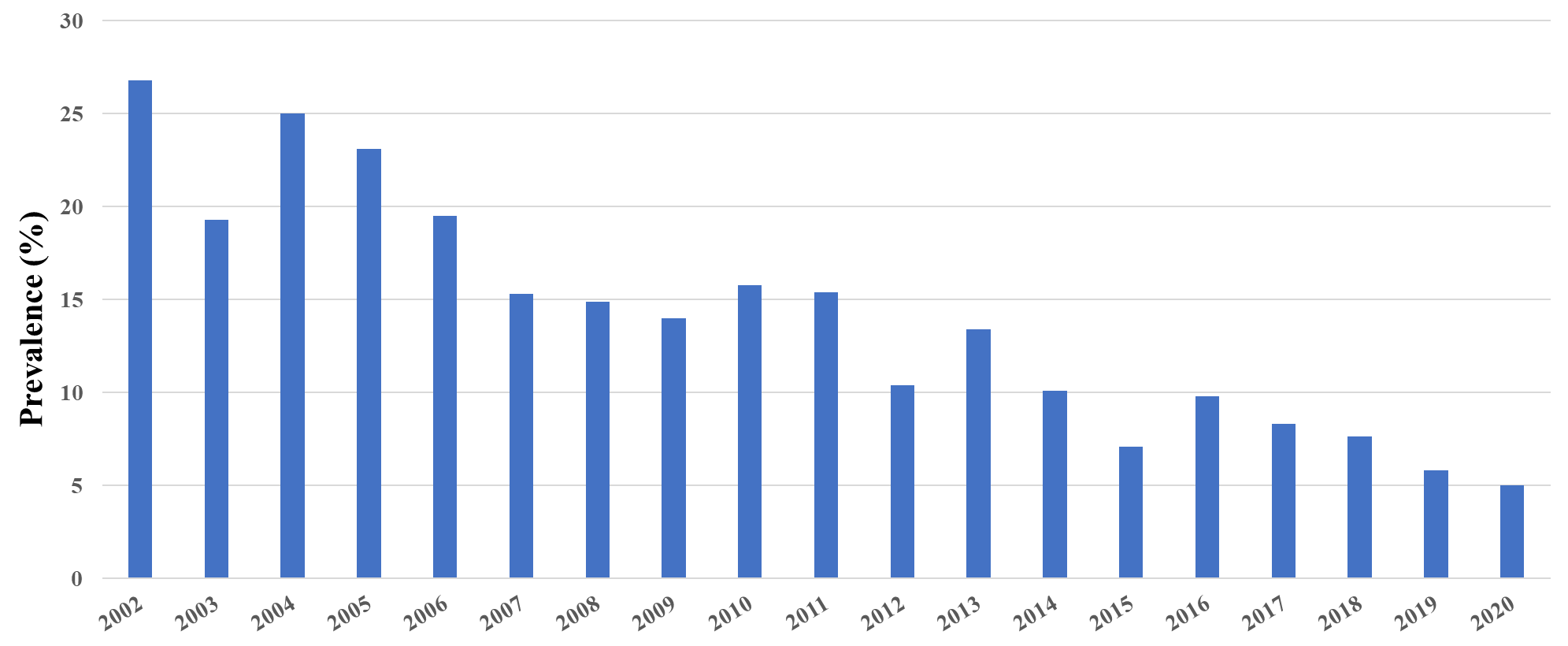


**Fig A. The overall prevalence of STH between 2002 and 2020.**

In 2017, this project set a roadmap for its goal of eliminating STH, defined as an STH prevalence in school-age children (SAC) and people covered by the Phufa project of less than 5% by 2026 (defined by the Thai Department of Disease Control). In 2015, the annual interventions of test and treat (TnT) and mass drug administration (MDA) were initiated. The current control intervention began with a screening test (the KK test) to detect helminth infections; any individuals who were found to have a helminth infection received selective treatment (the TnT intervention). Then, six months later, MDA was carried out for all SAC. For MDA treatment, albendazole (ALB), at a dosage of 200 mg, was given to all SAC. The prescribed amount is 400 mg (two tablets) to be taken as a single dose. The target populations were SAC in eight types of schools and participants in the Phufa project.

The eight types of schools were:

(A) Border patrol police (BPP) schools, comprising 218 schools with

an enrollment of approximately 25,946 children.

(B) Schools under the Office of the Basic Education Commission (OBEC), comprising 221 schools with an enrollment of approximately 47,499 children.

(C) Community learning centers of the Office of the Non-formal Education Commission, comprising 282 schools with an enrollment of approximately 7,873 children.

(D) Monastic schools under the National Office of Buddhism, comprising 69 schools with an enrollment of approximately 4,145 children.

(E) Private Islamic schools under the Office of the Private Education Commission, comprising 17 schools with an enrollment of approximately 13,427 children.

(F) Schools under the local government and child development centers, comprising 39 schools with an enrollment of approximately 2,040 children.

(G) Schools under the Bangkok Metropolitan Administration (BMA), comprising 25 schools with an enrollment of approximately 2,839 children.

(H) Rajaprajanugroh schools, comprising 43 schools with an enrollment of approximately 20,276 children.

For (I) the Phufa project, the selected areas were Bo Kluea and Chaloem Phra Kiat districts in Nan province.

Between 2015 and 2020, data from the survey of helminth infections conducted by this project indicated that the prevalence of helminth infections was between 5% and 10%, with no signs of further reductions. STH were the most commonly reported infections in this project. In 2017, the project set a roadmap for its goal of STH elimination, defined as a prevalence of less than 5%, by 2026. This project targets SAC who attends the eight types of schools and individuals living in Nan province who are covered by the Phufa project.

The numbers that make up the target populations and testing coverage are shown in Table A and Fig B. The project’s annual testing and giving treatment coverages, TnT, for the 6 years between 2015 and 2020 were 82.36%, 82.68%, 74.74%, 74.47%, 75.31%, and 66.61%, respectively. Three types of schools had more than 80% testing coverage over the 6-year period: the border patrol police (BPP) schools, schools under local government, and child development centers. The Phufa project had less than 60% testing coverage.

**Table A. Summary of the target populations and testing coverage.**

| Population type | 2015 | | 2016 | | 2017 | | 2018 | | 2019 | | 2020 | |
| --- | --- | --- | --- | --- | --- | --- | --- | --- | --- | --- | --- | --- |
|  | Total population | Number tested | Total population | Number tested | Total population | Number tested | Total population | Number tested | Total population | Number tested | Total population | Number tested |
| 1. Border patrol police (BPP) schools | 17209 | 15325 | 18052 | 16344 | 23768 | 21913 | 22713 | 20265 | 24871 | 22474 | 25946 | 20722 |
| 2. Schools under the Office of the Basic Education Commission (OBEC) | 28234 | 22638 | 27902 | 22865 | 36658 | 24814 | 35731 | 28207 | 47220 | 33248 | 47499 | 28031 |
| 3. Community learning centers of the Office of the Non-formal Education Commission | 3522 | 2441 | 8395 | 6845 | 7567 | 5847 | 7587 | 6392 | 7849 | 6858 | 7873 | 6051 |
| 4. Monastic schools under the National Office of Buddhism | 1782 | 1694 | 3037 | 2633 | 4783 | 4407 | 5560 | 4216 | 5037 | 4118 | 4145 | 3427 |
| 5. Private Islamic schools under the Office of the Private Education Commission | 4172 | 2769 | 8459 | 6215 | 10719 | 8781 | 9610 | 5853 | 11842 | 7445 | 13427 | 8824 |
| 6. Schools under local government and child development centers | 1237 | 11073 | 2528 | 2331 | 2059 | 1812 | 2110 | 1749 | 2042 | 1942 | 2040 | 1790 |
| 7. Schools under the Bangkok Metropolitan Administration (BMA) | 0 | 0 | 0 | 0 | 515 | 231 | 2839 | 1052 | 0 | 0 | 0 | 0 |
| 8. Rajaprajanugroh schools | 14110 | 11934 | 17548 | 13803 | 18700 | 13071 | 17839 | 12393 | 22740 | 18261 | 20276 | 15193 |
| Total school population | 70266 | 57874 | 85921 | 71036 | 104769 | 80876 | 103989 | 80127 | 121601 | 94346 | 121206 | 84038 |
| **School testing population coverage (%)** | **82.36** | | **82.68** | | **77.19** | | **77.05** | | **77.59** | | **69.33** | |
| 9. Phufa project | - | - | - | - | 13300 | 7364 | 9580 | 4448 | 15850 | 9169 | 17781 | 8541 |
| Total | 70266 | 557874 | 85921 | 71036 | 118069 | 88240 | 113569 | 84575 | 137451 | 103515 | 138987 | 92579 |
| **Testing coverage (%)** | **82.36** | | **82.68** | | **74.74** | | **74.47** | | **75.31** | | **66.61** | |

**
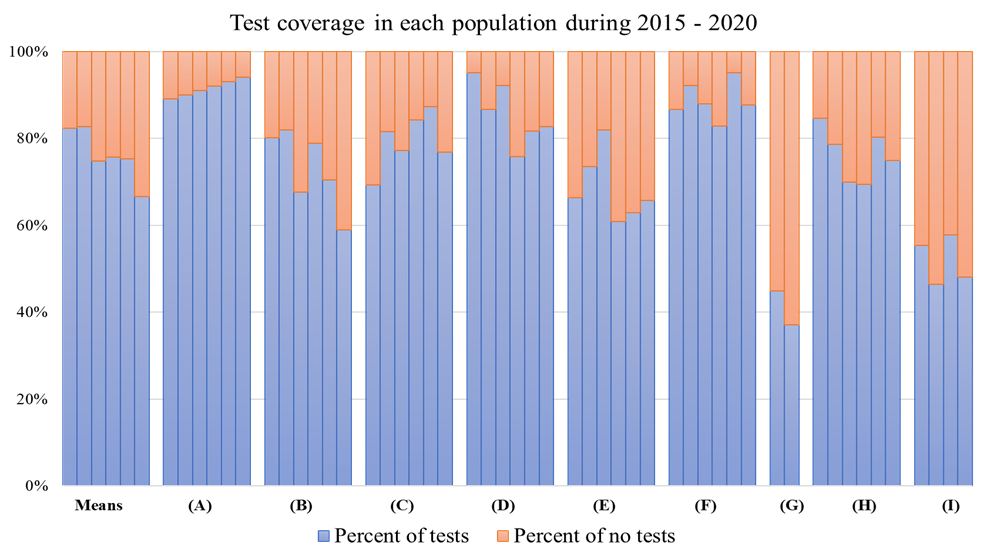
**

**Fig B. Testing coverage in each target population. (A) Border Patrol Police schools, (B) Schools under the Office of the Basic Education Commission, (C) Community learning centers of the Office of the Non-formal Education Commission, (D) Monastic schools under the National Office of Buddhism, (E) Private Islamic schools under the Office of the Private Education Commission, (F) Schools under the local government and child development centers, (G) Schools under the Bangkok Metropolitan Administration (BMA), (H) Rajaprajanugroh schools, and (I) the Phufa project.**

The overall annual prevalences of helminth infections from 2015 to 2020 were 6.74%, 9.41%, 7.37%, 7.62%, 5.82%, and 5.01%, respectively (Fig C). The infection rate of *A*. *lumbricoides* was highest during these 6 years, followed by *T*. *trichiura*, and then hookworms. The prevalence of *A*. *lumbricoides* infections was 3.10 to 5.81%; the prevalences of *T*. *trichiura* and hookworm infections were 1.49 to 2.27% and 0.28 to 0.99%, respectively. The trend in STH since 2015 was assessed based on school types.


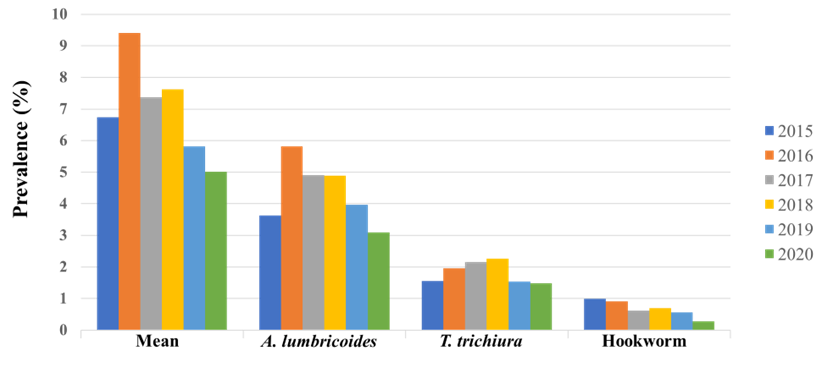
**.**

**Fig C. Helminth infections based on helminth spp.**

The overall prevalence showed variations among the types of schools. The prevalences in SAC can be categorized into three groups (Fig B):

- The helminth infection rates were highest in the community learning centers of the Office of the Non-formal Education Commission. The overall annual prevalences for the 6 years (2015 to 2020) were 43.42%, 45.73, 40.28%, 34.20%, 27.21%, and 25.65%, respectively. The infection rates of *A*. *lumbricoides* and *T*. *trichiura* ranged from 33.89% to 18.13% and 15.80% to 9.04%, respectively, while the infection rate of hookworms was less than 1% (Fig D).
- The helminth infection rates ranged between 5% and 10% in three school types: BPP schools, schools under OBEC, and schools under the local government and child development centers. In BPP schools, the overall annual prevalences for the 6 years (2015 to 2020) were 6.13%, 6.62%, 4.63%, 5.11%, 4.71%, and 3.99%, respectively. The infection rate of *A*. *lumbricoides* ranged between 3.61% and 2.40%, while the infection rates of *T*. *trichiura* and hookworms were less than 2%. In OBEC schools, the overall annual prevalences for the 6 years (2015 to 2020) were 6.09%, 7.16%, 6.98%, 7.43%, 5.32%, and 4.34%, respectively. The infection rate of *A*. *lumbricoides* ranged between 2.88% and 4.34%, while the infection rates of *T*. *trichiura* and hookworms were less than 2%, except that the *T*. *trichiura* infection rate in 2018 was 2.26%. For schools under the local government and child development centers, the overall annual prevalences for the 6 years (2015 to 2020) were 6.09%, 7.16%, 6.98%, 7.43%, 5.32%, and 4.34%, respectively (Fig D).
- The helminth infection rates were less than 5% in four types of schools: Private Islamic schools under the Office of the Private Education Commission, schools under the BMA, and Rajaprajanugroh schools.

Among species responsible for STH , the prevalence of *A*. *lumbricoides* was higher than that of *T*. *trichiura* and hookworms in four types of schools (Fig D): community learning centers of the Office of the Non-formal Education Commission, BPP schools, schools under OBEC, and schools under the local government and child development centers.

**
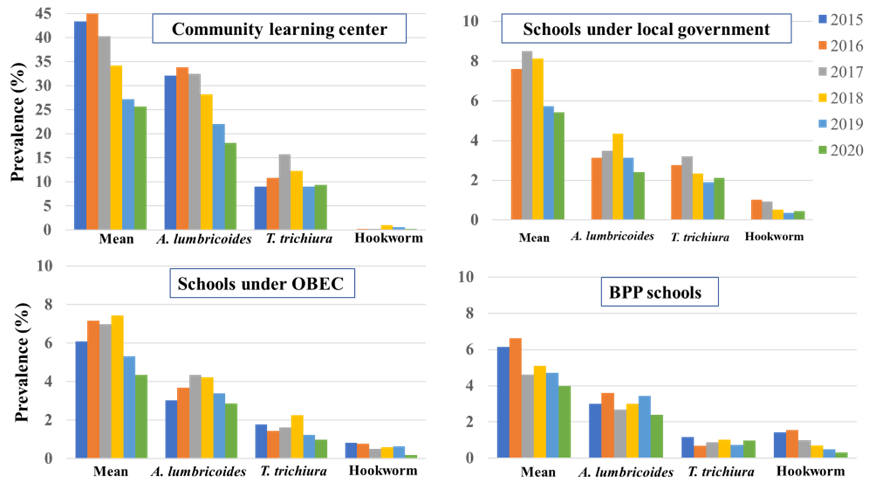
**

**Fig D. Helminth infections based on helminth spp.**

The overall number of STH declined between 2017 and 2026. However, when considering specific areas and school affiliations, it was found that SAC in the northern part of Thailand and schools located in highland areas where transportation is difficult, including those under the Office of Non-Formal and Informal Education, continued to exhibit a relatively high rates of STH infections. In contrast, schools in urban areas with more convenient transportation had a relatively low rate of STH infections.

It should be noted that this project had some limitations. These include individual-level issues, such as a lack of understanding about helminth surveillance; environmental problems, such as water shortages; and challenges in environmental and waste management. Importantly, in remote areas, there were difficulties in helminth screening and challenging transportation conditions, which resulted in delays in collecting or sending stool samples for testing.

STH infections among SAC in remote areas of Thailand exhibited distinctive characteristics. First, the overall prevalence of STH infections in SAC showed a decreasing trend from 2015 to 2020. Second, there were variations in STH rates among different areas and school types, with some schools demonstrating much higher rates, while others exhibited markedly lower rates. Third, the most common STH was caused by *A*. *lumbricoides*. To effectively eliminate STH, an optimized control strategy should be focused on different areas based on their prevalences.

*Data available*

**Table B. Prevalence of any STH infections between 2002 to 2020 in Thailand through the project: Controlling helminth infection in children and youth in remote areas**

| Years | Prevalence (%) |
| --- | --- |
| 2002 | 26.78 |
| 2003 | 19.30 |
| 2004 | 25.00 |
| 2005 | 23.10 |
| 2006 | 19.50 |
| 2007 | 15.30 |
| 2008 | 14.90 |
| 2009 | 14.00 |
| 2010 | 15.80 |
| 2011 | 15.40 |
| 2012 | 10.40 |
| 2013 | 13.40 |
| 2014 | 10.10 |
| 2015 | 7.10 |
| 2016 | 9.80 |
| 2017 | 8.34 |
| 2018 | 7.64 |
| 2019 | 5.82 |
| 2020 | 5.01 |

**References**

1. Bureau of General Communicable Diseases, Department of Disease Control. Controlling helminth infection in children and Youth in remote areas project. Ministry of Public Health, Thailand., 2019.

2. Annual report 2023 [Internet]. Ministry of Public Health, Thailand. 2024 [cited 11/02/2025]. Available from: <https://ddc.moph.go.th/uploads/ckeditor2/dcd/files/AnnualReport2023_DCD_2nd.pdf>.

3. Children and Youth Development Plan [Internet]. [cited 10/07/2024]. Available from: <https://www.psproject.org/wp-content/uploads/2021/03/child_dev_plan60_69.pdf>.
