# Supplementary material for: Mathematical modeling of control strategies for the elimination of soil-transmitted helminthiases in Thailand
Source: PLoS Negl Trop Dis. 2025 Aug 22;19(8):e0013435. doi: 10.1371/journal.pntd.0013435 (PMC12373168; doi:10.1371/journal.pntd.0013435)
Supplement: S3 Table — (S3_Table.DOCX) [file pntd.0013435.s003.docx]

**Mathematical modeling of control strategies for the elimination of soil-transmitted helminthiases in Thailand**

**Supporting information S3 Table**

**Table A:** Comparison of prevalence estimate data under the current intervention on different target populations.

**Table B:** Comparison of worm number estimate data under the current intervention on different target populations.

**Table C:** Comparison of prevalence estimate data under the biannual MDA intervention on different target populations at a coverage of 95%.

**Table D:** Comparison of worm number estimate data under the biannual MDA intervention on different target populations at a coverage of 95%.

**Table E:** Comparison of prevalence estimate data under the biannual TnT intervention on different target populations at a coverage of 95%.

**Table F:** Comparison of worm number estimate data under the biannual MDA intervention on different target populations at a coverage of 95%.

**Table A. Comparison of prevalence estimate data under the current intervention on different target populations.**

| Year | Current intervention on different target populations and coverages | | | | | | |
| --- | --- | --- | --- | --- | --- | --- | --- |
|  | SAC | SAC + adults (50%) | SAC + adults (80%) | SAC + PSAC (50%) | SAC + PSAC (80%) | SAC, adults (50%), PSAC (50%) | SAC, adults (80%), PSAC (80%) |
|  | Prevalence in PSAC (%) | | | | | | |
| 2024 | 6.91 | 6.91 | 6.91 | 6.91 | 6.91 | 6.91 | 6.91 |
| 2025 | 6.89 | 6.89 | 6.89 | 6.79 | 6.69 | 6.79 | 6.69 |
| 2026 | 6.88 | 6.87 | 6.86 | 6.74 | 6.59 | 6.73 | 6.56 |
| 2027 | 6.87 | 6.84 | 6.81 | 6.71 | 6.55 | 6.68 | 6.49 |
| 2028 | 6.86 | 6.82 | 6.77 | 6.70 | 6.54 | 6.65 | 6.44 |
| 2029 | 6.86 | 6.80 | 6.73 | 6.69 | 6.53 | 6.63 | 6.40 |
| 2030 | 6.85 | 6.78 | 6.70 | 6.69 | 6.52 | 6.61 | 6.37 |
| 2031 | 6.85 | 6.77 | 6.68 | 6.69 | 6.52 | 6.60 | 6.34 |
| 2032 | 6.85 | 6.76 | 6.66 | 6.68 | 6.52 | 6.59 | 6.32 |
| 2033 | 6.85 | 6.75 | 6.65 | 6.68 | 6.51 | 6.58 | 6.31 |
|  | Prevalence in SAC (%) | | | | | | |
| 2024 | 6.19 | 6.19 | 6.19 | 6.19 | 6.19 | 6.19 | 6.19 |
| 2025 | 6.17 | 6.17 | 6.17 | 6.17 | 6.17 | 6.17 | 6.17 |
| 2026 | 6.16 | 6.15 | 6.13 | 6.16 | 6.16 | 6.15 | 6.13 |
| 2027 | 6.15 | 6.12 | 6.09 | 6.15 | 6.15 | 6.12 | 6.08 |
| 2028 | 6.15 | 6.10 | 6.04 | 6.14 | 6.14 | 6.09 | 6.04 |
| 2029 | 6.14 | 6.08 | 6.01 | 6.14 | 6.13 | 6.07 | 6.00 |
| 2030 | 6.14 | 6.06 | 5.98 | 6.14 | 6.13 | 6.06 | 5.97 |
| 2031 | 6.14 | 6.05 | 5.96 | 6.13 | 6.13 | 6.05 | 5.95 |
| 2032 | 6.14 | 6.04 | 5.95 | 6.13 | 6.12 | 6.04 | 5.93 |
| 2033 | 6.13 | 6.04 | 5.93 | 6.13 | 6.12 | 6.03 | 5.92 |
|  | Prevalence in adults (%) | | | | | | |
| 2024 | 5.79 | 5.79 | 5.79% | 5.79% | 5.79 | 5.79 | 5.79 |
| 2025 | 5.77 | 5.67 | 5.57% | 5.77% | 5.77 | 5.67 | 5.57 |
| 2026 | 5.76 | 5.61 | 5.45% | 5.76% | 5.76 | 5.61 | 5.45 |
| 2027 | 5.75 | 5.57 | 5.38% | 5.75% | 5.74 | 5.56 | 5.37 |
| 2028 | 5.74 | 5.54 | 5.32% | 5.74% | 5.74 | 5.53 | 5.32 |
| 2029 | 5.74 | 5.51 | 5.29% | 5.73% | 5.73 | 5.51 | 5.28 |
| 2030 | 5.73 | 5.50 | 5.26% | 5.73% | 5.72 | 5.49 | 5.25 |
| 2031 | 5.73 | 5.49 | 5.24% | 5.72% | 5.72 | 5.48 | 5.22 |
| 2032 | 5.73 | 5.48 | 5.22% | 5.72% | 5.72 | 5.47 | 5.20 |
| 2033 | 5.73 | 5.47 | 5.21% | 5.72% | 5.71 | 5.46 | 5.19 |

**Table B. Comparison of worm number estimate data under the current intervention on different target populations.**

| Year | Current intervention on different target populations and coverages | | | | | | |
| --- | --- | --- | --- | --- | --- | --- | --- |
|  | SAC | SAC + adults (50%) | SAC + adults (80%) | SAC + PSAC (50%) | SAC + PSAC (80%) | SAC, adults (50%), PSAC (50%) | SAC, adults (80%), PSAC (80%) |
|  | Worm number in PSAC | | | | | | |
| 2024 | 7.365 | 7.365 | 7.365 | 7.365 | 7.365 | 7.365 | 7.365 |
| 2025 | 7.245 | 7.241 | 7.238 | 6.578 | 5.938 | 6.574 | 5.931 |
| 2026 | 7.158 | 7.075 | 6.996 | 6.232 | 5.398 | 6.156 | 5.264 |
| 2027 | 7.094 | 6.893 | 6.698 | 6.085 | 5.210 | 5.905 | 4.892 |
| 2028 | 7.047 | 6.731 | 6.426 | 6.011 | 5.129 | 5.732 | 4.644 |
| 2029 | 7.011 | 6.600 | 6.206 | 5.968 | 5.085 | 5.608 | 4.468 |
| 2030 | 6.985 | 6.499 | 6.034 | 5.940 | 5.058 | 5.516 | 4.338 |
| 2031 | 6.966 | 6.422 | 5.904 | 5.920 | 5.039 | 5.448 | 4.240 |
| 2032 | 6.952 | 6.364 | 5.805 | 5.905 | 5.025 | 5.397 | 4.167 |
| 2033 | 6.941 | 6.320 | 5.729 | 5.895 | 5.014 | 5.359 | 4.111 |
|  | Worm number in SAC | | | | | | |
| 2024 | 3.651 | 3.651 | 3.651 | 3.651 | 3.651 | 3.651 | 3.651 |
| 2025 | 3.598 | 3.595 | 3.594 | 3.598 | 3.598 | 3.595 | 3.593 |
| 2026 | 3.559 | 3.511 | 3.464 | 3.556 | 3.553 | 3.507 | 3.457 |
| 2027 | 3.531 | 3.418 | 3.309 | 3.523 | 3.515 | 3.410 | 3.292 |
| 2028 | 3.510 | 3.339 | 3.174 | 3.498 | 3.486 | 3.327 | 3.149 |
| 2029 | 3.494 | 3.278 | 3.070 | 3.479 | 3.464 | 3.262 | 3.037 |
| 2030 | 3.482 | 3.231 | 2.991 | 3.465 | 3.448 | 3.212 | 2.952 |
| 2031 | 3.474 | 3.196 | 2.931 | 3.454 | 3.436 | 3.175 | 2.888 |
| 2032 | 3.467 | 3.169 | 2.886 | 3.447 | 3.426 | 3.147 | 2.839 |
| 2033 | 3.462 | 3.150 | 2.852 | 3.441 | 3.420 | 3.126 | 2.801 |
|  | Worm number in adults | | | | | | |
| 2024 | 2.472 | 2.472 | 2.472 | 2.472 | 2.472 | 2.472 | 2.472 |
| 2025 | 2.432 | 2.213 | 2.000 | 2.432 | 2.432 | 2.213 | 2.000 |
| 2026 | 2.403 | 2.076 | 1.780 | 2.401 | 2.399 | 2.074 | 1.777 |
| 2027 | 2.381 | 1.994 | 1.660 | 2.377 | 2.372 | 1.990 | 1.652 |
| 2028 | 2.366 | 1.938 | 1.580 | 2.358 | 2.351 | 1.932 | 1.568 |
| 2029 | 2.354 | 1.899 | 1.524 | 2.344 | 2.335 | 1.890 | 1.508 |
| 2030 | 2.345 | 1.869 | 1.483 | 2.334 | 2.322 | 1.859 | 1.464 |
| 2031 | 2.338 | 1.848 | 1.453 | 2.326 | 2.313 | 1.836 | 1.432 |
| 2032 | 2.334 | 1.832 | 1.430 | 2.320 | 2.307 | 1.819 | 1.407 |
| 2033 | 2.330 | 1.819 | 1.413 | 2.316 | 2.301 | 1.806 | 1.388 |
|  | Worm number in environment | | | | | | |
| 2024 | 3.670 | 3.670 | 3.670 | 3.670 | 3.670 | 3.670 | 3.670 |
| 2025 | 3.629 | 3.618 | 3.611 | 3.628 | 3.628 | 3.617 | 3.610 |
| 2026 | 3.598 | 3.512 | 3.429 | 3.592 | 3.586 | 3.506 | 3.417 |
| 2027 | 3.576 | 3.423 | 3.276 | 3.565 | 3.555 | 3.412 | 3.254 |
| 2028 | 3.559 | 3.355 | 3.161 | 3.545 | 3.531 | 3.341 | 3.131 |
| 2029 | 3.547 | 3.305 | 3.076 | 3.530 | 3.513 | 3.287 | 3.039 |
| 2030 | 3.537 | 3.267 | 3.012 | 3.518 | 3.500 | 3.247 | 2.970 |
| 2031 | 3.530 | 3.239 | 2.964 | 3.510 | 3.491 | 3.217 | 2.918 |
| 2032 | 3.525 | 3.218 | 2.927 | 3.504 | 3.483 | 3.195 | 2.878 |
| 2033 | 3.521 | 3.202 | 2.899 | 3.499 | 3.478 | 3.178 | 2.847 |

**Table C. Comparison of prevalence estimate data under the biannual MDA intervention on different target populations at a coverage of 95%.**

| Year | Comparison between the current intervention and the biannual MDA intervention on different target populations and coverages | | | | | | | |
| --- | --- | --- | --- | --- | --- | --- | --- | --- |
|  | Current | SAC | SAC + adults (50%) | SAC + adults (80%) | SAC + PSAC (50%) | SAC + PSAC (80%) | SAC, adults (50%), PSAC (50%) | SAC, adults (80%), PSAC (80%) |
|  | Prevalence in PSAC (%) | | | | | | | |
| 2024 | 6.91 | 6.91 | 6.91 | 6.91 | 6.91 | 6.91 | 6.91 | 6.91 |
| 2025 | 6.89 | 6.88 | 6.85 | 6.83 | 6.09 | 5.41 | 6.04 | 5.29 |
| 2026 | 6.88 | 6.83 | 6.68 | 6.56 | 5.87 | 5.23 | 5.64 | 4.71 |
| 2027 | 6.87 | 6.79 | 6.46 | 6.24 | 5.80 | 5.17 | 5.33 | 4.24 |
| 2028 | 6.86 | 6.75 | 6.24 | 5.90 | 5.76 | 5.13 | 5.07 | 3.80 |
| 2029 | 6.86 | 6.73 | 6.04 | 5.59 | 5.73 | 5.09 | 4.83 | 3.37 |
| 2030 | 6.85 | 6.70 | 5.86 | 5.29 | 5.70 | 5.07 | 4.61 | 2.94 |
| 2031 | 6.85 | 6.69 | 5.70 | 5.02 | 5.68 | 5.05 | 4.41 | 2.51 |
| 2032 | 6.85 | 6.68 | 5.55 | 4.75 | 5.66 | 5.02 | 4.20 | 2.06 |
| 2033 | 6.85 | 6.66 | 5.41 | 4.50 | 5.65 | 5.01 | 4.00 | 1.60 |
|  | Prevalence in SAC (%) | | | | | | | |
| 2024 | 6.19 | 6.19 | 6.19 | 6.19 | 6.19 | 6.19 | 6.19 | 6.19 |
| 2025 | 6.17 | 4.72 | 4.64 | 4.56 | 4.72 | 4.71 | 4.63 | 4.55 |
| 2026 | 6.16 | 4.64 | 4.34 | 4.12 | 4.62 | 4.61 | 4.31 | 4.05 |
| 2027 | 6.15 | 4.61 | 4.10 | 3.74 | 4.57 | 4.55 | 4.03 | 3.58 |
| 2028 | 6.15 | 4.58 | 3.89 | 3.42 | 4.54 | 4.51 | 3.78 | 3.15 |
| 2029 | 6.14 | 4.56 | 3.71 | 3.13 | 4.51 | 4.48 | 3.56 | 2.73 |
| 2030 | 6.14 | 4.54 | 3.55 | 2.88 | 4.48 | 4.45 | 3.35 | 2.32 |
| 2031 | 6.14 | 4.53 | 3.41 | 2.63 | 4.47 | 4.43 | 3.15 | 1.91 |
| 2032 | 6.14 | 4.52 | 3.27 | 2.40 | 4.45 | 4.41 | 2.95 | 1.50 |
| 2033 | 6.13 | 4.51 | 3.15 | 2.18 | 4.44 | 4.39 | 2.77 | 1.11 |
|  | Prevalence in adults (%) | | | | | | | |
| 2024 | 5.79 | 5.79 | 5.79 | 5.79 | 5.79 | 5.79 | 5.79 | 5.79 |
| 2025 | 5.77 | 5.76 | 4.92 | 4.17 | 5.76 | 5.75 | 4.92 | 4.16 |
| 2026 | 5.76 | 5.71 | 4.53 | 3.65 | 5.70 | 5.69 | 4.51 | 3.59 |
| 2027 | 5.75 | 5.67 | 4.26 | 3.27 | 5.64 | 5.63 | 4.20 | 3.13 |
| 2028 | 5.74 | 5.63 | 4.03 | 2.95 | 5.60 | 5.57 | 3.94 | 2.70 |
| 2029 | 5.74 | 5.60 | 3.84 | 2.67 | 5.56 | 5.53 | 3.70 | 2.29 |
| 2030 | 5.73 | 5.58 | 3.68 | 2.41 | 5.53 | 5.50 | 3.49 | 1.90 |
| 2031 | 5.73 | 5.57 | 3.53 | 2.18 | 5.51 | 5.47 | 3.29 | 1.52 |
| 2032 | 5.73 | 5.55 | 3.39 | 1.96 | 5.49 | 5.45 | 3.09 | 1.16 |
| 2033 | 5.73 | 5.54 | 3.26 | 1.75 | 5.47 | 5.43 | 2.89 | 0.82 |

**Table D. Comparison of worm number estimate data under the biannual MDA intervention on different target populations at a coverage of 95%.**

| Year | Comparison between the current intervention and the biannual MDA intervention on different target populations and coverages | | | | | | | |
| --- | --- | --- | --- | --- | --- | --- | --- | --- |
|  | Current | SAC | SAC + adults (50%) | SAC + adults (80%) | SAC + PSAC (50%) | SAC + PSAC (80%) | SAC, adults (50%), PSAC (50%) | SAC, adults (80%), PSAC (80%) |
|  | Worm number in PSAC | | | | | | | |
| 2024 | 7.365 | 7.365 | 7.365 | 7.365 | 7.365 | 7.365 | 7.365 | 7.365 |
| 2025 | 7.245 | 7.159 | 6.967 | 6.801 | 3.312 | 1.722 | 3.172 | 1.528 |
| 2026 | 7.158 | 6.831 | 5.875 | 5.260 | 2.690 | 1.447 | 2.135 | 0.877 |
| 2027 | 7.094 | 6.547 | 4.749 | 3.822 | 2.505 | 1.364 | 1.591 | 0.555 |
| 2028 | 7.047 | 6.328 | 3.841 | 2.766 | 2.403 | 1.308 | 1.234 | 0.360 |
| 2029 | 7.011 | 6.162 | 3.157 | 2.035 | 2.331 | 1.265 | 0.981 | 0.236 |
| 2030 | 6.985 | 6.035 | 2.648 | 1.530 | 2.275 | 1.232 | 0.793 | 0.154 |
| 2031 | 6.966 | 5.939 | 2.262 | 1.172 | 2.232 | 1.206 | 0.650 | 0.099 |
| 2032 | 6.952 | 5.863 | 1.960 | 0.910 | 2.187 | 1.180 | 0.533 | 0.062 |
| 2033 | 6.941 | 5.804 | 1.719 | 0.713 | 2.158 | 1.163 | 0.441 | 0.037 |
|  | Worm number in SAC | | | | | | | |
| 2024 | 3.651 | 3.651 | 3.651 | 3.651 | 3.651 | 3.651 | 3.651 | 3.651 |
| 2025 | 3.598 | 0.882 | 0.814 | 0.758 | 0.877 | 0.872 | 0.809 | 0.747 |
| 2026 | 3.559 | 0.819 | 0.613 | 0.492 | 0.803 | 0.790 | 0.595 | 0.459 |
| 2027 | 3.531 | 0.790 | 0.483 | 0.342 | 0.765 | 0.748 | 0.454 | 0.293 |
| 2028 | 3.510 | 0.769 | 0.394 | 0.249 | 0.738 | 0.718 | 0.356 | 0.191 |
| 2029 | 3.494 | 0.753 | 0.331 | 0.188 | 0.718 | 0.696 | 0.285 | 0.125 |
| 2030 | 3.482 | 0.741 | 0.283 | 0.145 | 0.702 | 0.678 | 0.232 | 0.082 |
| 2031 | 3.474 | 0.732 | 0.246 | 0.114 | 0.689 | 0.664 | 0.191 | 0.052 |
| 2032 | 3.467 | 0.723 | 0.216 | 0.089 | 0.678 | 0.651 | 0.157 | 0.033 |
| 2033 | 3.462 | 0.718 | 0.191 | 0.070 | 0.670 | 0.642 | 0.130 | 0.019 |
|  | Worm number in adults | | | | | | | |
| 2024 | 2.472 | 2.472 | 2.472 | 2.472 | 2.472 | 2.472 | 2.472 | 2.472 |
| 2025 | 2.432 | 2.403 | 1.068 | 0.519 | 2.399 | 2.393 | 1.065 | 0.513 |
| 2026 | 2.403 | 2.293 | 0.732 | 0.313 | 2.268 | 2.247 | 0.717 | 0.294 |
| 2027 | 2.381 | 2.198 | 0.562 | 0.215 | 2.150 | 2.115 | 0.534 | 0.186 |
| 2028 | 2.366 | 2.124 | 0.453 | 0.156 | 2.056 | 2.011 | 0.414 | 0.121 |
| 2029 | 2.354 | 2.068 | 0.376 | 0.117 | 1.984 | 1.931 | 0.329 | 0.079 |
| 2030 | 2.345 | 2.026 | 0.320 | 0.090 | 1.929 | 1.869 | 0.266 | 0.052 |
| 2031 | 2.338 | 1.994 | 0.277 | 0.071 | 1.886 | 1.821 | 0.218 | 0.033 |
| 2032 | 2.334 | 1.968 | 0.241 | 0.055 | 1.852 | 1.783 | 0.179 | 0.021 |
| 2033 | 2.330 | 1.948 | 0.213 | 0.044 | 1.825 | 1.752 | 0.148 | 0.012 |
|  | Worm number in environment | | | | | | | |
| 2024 | 3.670 | 3.670 | 3.670 | 3.670 | 3.670 | 3.670 | 3.670 | 3.670 |
| 2025 | 3.629 | 3.467 | 3.083 | 2.783 | 3.438 | 3.408 | 3.053 | 2.722 |
| 2026 | 3.598 | 3.298 | 2.366 | 1.847 | 3.224 | 3.167 | 2.284 | 1.700 |
| 2027 | 3.576 | 3.187 | 1.877 | 1.296 | 3.081 | 3.009 | 1.751 | 1.088 |
| 2028 | 3.559 | 3.107 | 1.540 | 0.951 | 2.976 | 2.893 | 1.378 | 0.709 |
| 2029 | 3.547 | 3.046 | 1.296 | 0.721 | 2.897 | 2.804 | 1.106 | 0.465 |
| 2030 | 3.537 | 2.999 | 1.113 | 0.558 | 2.835 | 2.735 | 0.902 | 0.303 |
| 2031 | 3.530 | 2.963 | 0.970 | 0.438 | 2.786 | 2.680 | 0.742 | 0.194 |
| 2032 | 3.525 | 2.934 | 0.853 | 0.345 | 2.746 | 2.634 | 0.613 | 0.120 |
| 2033 | 3.521 | 2.911 | 0.757 | 0.272 | 2.714 | 2.598 | 0.507 | 0.070 |

**Table E. Comparison of prevalence estimate data under the biannual TnT intervention on the different target populations at a coverage of 95%.**

| Year | Comparison between the current intervention and the biannual TnT intervention on different target populations and coverages | | | | | | | |
| --- | --- | --- | --- | --- | --- | --- | --- | --- |
|  | Current | SAC | SAC + adults (50%) | SAC + adults (80%) | SAC + PSAC (50%) | SAC + PSAC (80%) | SAC, adults (50%), PSAC (50%) | SAC, adults (80%), PSAC (80%) |
|  | Prevalence in PSAC (%) | | | | | | | |
| 2024 | 6.91 | 6.91 | 6.91 | 6.91 | 6.91 | 6.91 | 6.91 | 6.91 |
| 2025 | 6.89 | 6.89 | 6.89 | 6.89 | 6.86 | 6.83 | 6.85 | 6.83 |
| 2026 | 6.88 | 6.88 | 6.88 | 6.87 | 6.83 | 6.80 | 6.83 | 6.80 |
| 2027 | 6.87 | 6.88 | 6.87 | 6.86 | 6.83 | 6.79 | 6.81 | 6.78 |
| 2028 | 6.86 | 6.88 | 6.86 | 6.85 | 6.82 | 6.79 | 6.81 | 6.76 |
| 2029 | 6.86 | 6.88 | 6.86 | 6.85 | 6.82 | 6.79 | 6.80 | 6.75 |
| 2030 | 6.85 | 6.88 | 6.85 | 6.84 | 6.82 | 6.79 | 6.80 | 6.75 |
| 2031 | 6.85 | 6.88 | 6.85 | 6.84 | 6.82 | 6.79 | 6.79 | 6.74 |
| 2032 | 6.85 | 6.88 | 6.85 | 6.83 | 6.82 | 6.79 | 6.79 | 6.74 |
| 2033 | 6.85 | 6.88 | 6.85 | 6.83 | 6.82 | 6.79 | 6.79 | 6.74 |
|  | Prevalence in SAC (%) | | | | | | | |
| 2024 | 6.19 | 6.19 | 6.19 | 6.19 | 6.19 | 6.19 | 6.19 | 6.19 |
| 2025 | 6.17 | 6.42 | 6.42 | 6.42 | 6.42 | 6.42 | 6.42 | 6.42 |
| 2026 | 6.16 | 6.50 | 6.49 | 6.49 | 6.50 | 6.50 | 6.49 | 6.49 |
| 2027 | 6.15 | 6.53 | 6.51 | 6.51 | 6.52 | 6.52 | 6.51 | 6.50 |
| 2028 | 6.15 | 6.53 | 6.52 | 6.51 | 6.53 | 6.53 | 6.52 | 6.50 |
| 2029 | 6.14 | 6.54 | 6.52 | 6.50 | 6.54 | 6.53 | 6.51 | 6.50 |
| 2030 | 6.14 | 6.54 | 6.51 | 6.50 | 6.54 | 6.54 | 6.51 | 6.50 |
| 2031 | 6.14 | 6.54 | 6.51 | 6.50 | 6.54 | 6.54 | 6.51 | 6.49 |
| 2032 | 6.14 | 6.54 | 6.51 | 6.49 | 6.54 | 6.54 | 6.51 | 6.49 |
| 2033 | 6.13 | 6.54 | 6.51 | 6.49 | 6.54 | 6.53 | 6.51 | 6.49 |
|  | Prevalence in adults (%) | | | | | | | |
| 2024 | 5.79 | 5.79 | 5.79 | 5.79 | 5.79 | 5.79 | 5.79 | 5.79 |
| 2025 | 5.77 | 5.77 | 5.74 | 5.72 | 5.77 | 5.77 | 5.74 | 5.72 |
| 2026 | 5.76 | 5.76 | 5.71 | 5.69 | 5.76 | 5.76 | 5.71 | 5.69 |
| 2027 | 5.75 | 5.76 | 5.70 | 5.67 | 5.76 | 5.76 | 5.70 | 5.67 |
| 2028 | 5.74 | 5.76 | 5.69 | 5.66 | 5.76 | 5.76 | 5.69 | 5.65 |
| 2029 | 5.74 | 5.76 | 5.69 | 5.65 | 5.76 | 5.75 | 5.69 | 5.65 |
| 2030 | 5.73 | 5.76 | 5.69 | 5.64 | 5.76 | 5.75 | 5.68 | 5.64 |
| 2031 | 5.73 | 5.76 | 5.68 | 5.64 | 5.76 | 5.75 | 5.68 | 5.64 |
| 2032 | 5.73 | 5.76 | 5.68 | 5.64 | 5.76 | 5.75 | 5.68 | 5.63 |
| 2033 | 5.73 | 5.76 | 5.68 | 5.63 | 5.76 | 5.75 | 5.68 | 5.63 |

**Table F. Comparison of worm number estimate data under the biannual MDA intervention on different target populations at a coverage of 95%.**

| Year | Comparison between the current intervention and the biannual TnT intervention on different target populations and coverages | | | | | | | |
| --- | --- | --- | --- | --- | --- | --- | --- | --- |
|  | Current | SAC | SAC + adults (50%) | SAC + adults (80%) | SAC + PSAC (50%) | SAC + PSAC (80%) | SAC, adults (50%), PSAC (50%) | SAC, adults (80%), PSAC (80%) |
|  | Worm number in PSAC | | | | | | | |
| 2024 | 7.365 | 7.365 | 7.365 | 7.365 | 7.365 | 7.365 | 7.365 | 7.365 |
| 2025 | 7.245 | 7.245 | 7.237 | 7.233 | 6.994 | 6.846 | 6.987 | 6.835 |
| 2026 | 7.158 | 7.185 | 7.145 | 7.122 | 6.848 | 6.655 | 6.810 | 6.594 |
| 2027 | 7.094 | 7.164 | 7.084 | 7.035 | 6.796 | 6.588 | 6.719 | 6.467 |
| 2028 | 7.047 | 7.158 | 7.040 | 6.970 | 6.778 | 6.564 | 6.666 | 6.389 |
| 2029 | 7.011 | 7.157 | 7.009 | 6.921 | 6.772 | 6.556 | 6.631 | 6.338 |
| 2030 | 6.985 | 7.158 | 6.986 | 6.885 | 6.770 | 6.552 | 6.607 | 6.301 |
| 2031 | 6.966 | 7.158 | 6.969 | 6.858 | 6.769 | 6.550 | 6.590 | 6.275 |
| 2032 | 6.952 | 7.159 | 6.957 | 6.838 | 6.765 | 6.545 | 6.574 | 6.251 |
| 2033 | 6.941 | 7.159 | 6.947 | 6.822 | 6.764 | 6.543 | 6.563 | 6.235 |
|  | Worm number in SAC | | | | | | | |
| 2024 | 3.651 | 3.651 | 3.651 | 3.651 | 3.651 | 3.651 | 3.651 | 3.651 |
| 2025 | 3.598 | 4.563 | 4.558 | 4.554 | 4.563 | 4.563 | 4.557 | 4.554 |
| 2026 | 3.559 | 4.939 | 4.909 | 4.892 | 4.937 | 4.935 | 4.907 | 4.888 |
| 2027 | 3.531 | 5.066 | 5.007 | 4.972 | 5.061 | 5.058 | 5.002 | 4.964 |
| 2028 | 3.510 | 5.110 | 5.024 | 4.973 | 5.103 | 5.099 | 5.018 | 4.963 |
| 2029 | 3.494 | 5.126 | 5.019 | 4.956 | 5.117 | 5.112 | 5.010 | 4.942 |
| 2030 | 3.482 | 5.132 | 5.009 | 4.936 | 5.122 | 5.116 | 4.999 | 4.920 |
| 2031 | 3.474 | 5.134 | 4.999 | 4.920 | 5.123 | 5.117 | 4.988 | 4.902 |
| 2032 | 3.467 | 5.132 | 4.988 | 4.903 | 5.121 | 5.114 | 4.976 | 4.884 |
| 2033 | 3.462 | 5.131 | 4.980 | 4.891 | 5.119 | 5.112 | 4.968 | 4.872 |
|  | Worm number in adult | | | | | | | |
| 2024 | 2.472 | 2.472 | 2.472 | 2.472 | 2.472 | 2.472 | 2.472 | 2.472 |
| 2025 | 2.432 | 2.432 | 2.359 | 2.316 | 2.432 | 2.432 | 2.359 | 2.316 |
| 2026 | 2.403 | 2.412 | 2.305 | 2.243 | 2.411 | 2.410 | 2.304 | 2.241 |
| 2027 | 2.381 | 2.405 | 2.277 | 2.204 | 2.403 | 2.402 | 2.275 | 2.200 |
| 2028 | 2.366 | 2.403 | 2.260 | 2.179 | 2.400 | 2.398 | 2.257 | 2.175 |
| 2029 | 2.354 | 2.403 | 2.249 | 2.163 | 2.399 | 2.396 | 2.246 | 2.157 |
| 2030 | 2.345 | 2.403 | 2.242 | 2.152 | 2.398 | 2.396 | 2.237 | 2.145 |
| 2031 | 2.338 | 2.403 | 2.236 | 2.144 | 2.398 | 2.395 | 2.232 | 2.136 |
| 2032 | 2.334 | 2.403 | 2.232 | 2.136 | 2.398 | 2.395 | 2.227 | 2.128 |
| 2033 | 2.330 | 2.403 | 2.228 | 2.131 | 2.398 | 2.394 | 2.223 | 2.122 |
|  | Worm number in environment | | | | | | | |
| 2024 | 3.670 | 3.670 | 3.670 | 3.670 | 3.670 | 3.670 | 3.670 | 3.670 |
| 2025 | 3.629 | 3.628 | 3.613 | 3.604 | 3.627 | 3.626 | 3.612 | 3.602 |
| 2026 | 3.598 | 3.626 | 3.585 | 3.561 | 3.623 | 3.621 | 3.582 | 3.556 |
| 2027 | 3.576 | 3.627 | 3.566 | 3.529 | 3.622 | 3.619 | 3.561 | 3.521 |
| 2028 | 3.559 | 3.628 | 3.551 | 3.505 | 3.622 | 3.619 | 3.545 | 3.495 |
| 2029 | 3.547 | 3.629 | 3.540 | 3.488 | 3.622 | 3.618 | 3.533 | 3.476 |
| 2030 | 3.537 | 3.630 | 3.532 | 3.475 | 3.622 | 3.618 | 3.525 | 3.462 |
| 2031 | 3.530 | 3.630 | 3.526 | 3.465 | 3.622 | 3.617 | 3.518 | 3.452 |
| 2032 | 3.525 | 3.630 | 3.522 | 3.458 | 3.622 | 3.617 | 3.513 | 3.444 |
| 2033 | 3.521 | 3.631 | 3.518 | 3.452 | 3.622 | 3.616 | 3.509 | 3.437 |
