# Supplementary material for: Mathematical modeling of control strategies for the elimination of soil-transmitted helminthiases in Thailand
Source: PLoS Negl Trop Dis. 2025 Aug 22;19(8):e0013435. doi: 10.1371/journal.pntd.0013435 (PMC12373168; doi:10.1371/journal.pntd.0013435)
Supplement: S4 Text — (S4_Text.DOCX) [file pntd.0013435.s004.docx]

**Mathematical modeling of control strategies for the elimination of soil-transmitted helminthiases in Thailand**

**Supporting information S4 Text**

#install required package

install.packages("deSolve")

install.packages("ggplot2")

install.packages("readr")

install.packages("ggpubr")

install.packages("writexl")

library (deSolve)

library (ggplot2)

library (readr)

library (ggpubr)

library (writexl)

##Define model parameters

##Helminth population (L,Mp,Ma,Mc) - worm number

#Parameters list for model----

#beta_p - Infection transmission rate (AL) among PSAC(p)

#beta_c - Infection transmission rate (AL) among SAC(c)

#beta_a - Infection transmission rate (AL) among adults(a)

#np - Proportion of PSAC in the population

#nc - Proportion of SAC in the population

#na - Proportion of adults in the population

#alpha_p - Relative contributions of PSAC to the environment

#alpha_c - Relative contributions of SAC to the environment

#alpha_a - Relative contributions of adults to the environment

#mu_W - Average life span of the mature worm in the host (1/mu_W = 365 days)

#mu_L - Average life span of free-living infectious materials (1/mu_L = 84 days)

#gamma - Strength of density dependence of worm egg production

#kcon - Aggregation parameter

#kin_1 - Aggregation parameter_1

#kin_2 - Aggregation parameter_2

#kin_3 - Aggregation parameter_3

#kin_4 - Aggregation parameter_4

#Calculation parameters in model

#psi_p - mating probability factor for AL among PSAC(p)

#psi_c - mating probability factor for AL among SAC(c)

#psi_a - mating probability factor for AL among adults(a)

#fp - mean egg production rate from host(PSAC) with mean worm burden

#fc - mean egg production rate from host(SAC) with mean worm burden

#fa - mean egg production rate from host(Adults) with mean worm burden

## parameters for test and treat(TnT) intervention

## intervention - day

#kato_sen - Kato test sensitivity

#kato_spec - Kato test specificity

#ProTnT_p - Proportion of testing by kato per TnT round

#ProTnT_c - Proportion of testing by kato per TnT round

#ProTnT_a - Proportion of testing by kato per TnT round

#h_p - drug efficacy

#h_c - drug efficacy

#h_a - drug efficacy

#campaigndays_TnT - time or duration of TnT intervention (days)

#time_interv_TnT - time when TnT intervention start (days)

#times_per_year_TnT - #times (days) per year of TnT (days),frequency of TnT per year

## Calculation TnT parameters

#TnTp,TnTc,TnTa - the number of infected individuals in each population who are detected and enter the treatment process

#TnTp <- (1-(1+(Mp/k))^-k)*kato_sen*ProTnT_p

#TnTc <- (1-(1+(Mc/k))^-k)*kato_sen*ProTnT_c

#TnTa <- (1-(1+(Ma/k))^-k)*kato_sen*ProTnT_a

#TnTp_rate <- (-log(1-(TnTp*h_p))/campaigndays_TnT)

#TnTc_rate <- (-log(1-(TnTc*h_c))/campaigndays_TnT)

#TnTa_rate <- (-log(1-(TnTa*h_a))/campaigndays_TnT)

#TnTp_p - TnT effect in PSAC on specific time

#TnTp_c - TnT effect in SAC on specific time

#TnTp_a - TnT effect in Adults on specific time

## parameters for MDA

## intervention - day

#gMDA_p - MDA coverage in PSAC (proportion,between 0-1)

#gMDA_c - MDA coverage in SAC (proportion,between 0-1)

#gMDA_a - MDA coverage in adults (proportion,between 0-1)

#campaigndays_MDA_p - time or duration of MDA treatment (days)

#campaigndays_MDA_c - time or duration of MDA treatment (days)

#campaigndays_MDA_a - time or duration of MDA treatment (days)

#time_interv_MDA_p - time when MDA start in days (days)

#time_interv_MDA_c - time when MDA start in days (days)

#time_interv_MDA_a - time when MDA start in days (days)

#times_per_year_MDA - #times (days) per year of MDA (days), frequency of MDA per year

## Calculation MDA parameters

#MDArate_i <- (-log(1-(gMDA_p*h_p))/campaigndays_MDA_p)

#MDArate_i <- (-log(1-(gMDA_p*h_p))/campaigndays_MDA_p)

#MDArate_i <- (-log(1-(gMDA_p*h_p))/campaigndays_MDA_p)

#MDAcampaign_p - effect of MDA on PSAC

#MDAcampaign_c - effect of MDA on SAC

#MDAcampaign_a - effect of MDA on adults

#############################

## Define initial state variables

initMp <- 1 #Mean worm in PSAC

initMc <- 1 #Mean worm in SAC

initMa <- 1 #Mean worm in adults

initL <- 2 #Larva in environment

state = c ( Mp = initMp, Mc = initMc, Ma = initMa,L = initL)

## Times

## Set time to run model

time_start <- 0

time_stop <- 70 #year

deltat <- 1/365 #year or 1/365(day)

## times = seq (0, 365*10, by=1)

times <- seq(time_start , time_stop , by = deltat)

## Define model parameters

parameters_1 <-c(

beta_p <- 1.972, #Infection transmission rate (AL) among PSAC(p)

beta_c <- 1.536, #Infection transmission rate (AL) among SAC(c)

beta_a <- 0.662, #Infection transmission rate (AL) among adults(a)

np <- 0.06, #Proportion of PSAC in the population

nc <- 0.14, #Proportion of SAC in the population

na <- 0.80, #Proportion of adults in the population

alpha_p <- 2.768, #Relative contributions of PSAC to the environment

alpha_c <- 2.726, #Relative contributions of SAC to the environment

alpha_a <- 2.675, #Relative contributions of adults to the environment

mu_W <- 1, #Average life span of the mature worm in the host (1/mu_W = 365 days)

mu_L <- 84/365, #Average life span of free-living infectious materials (1/mu_L = 84 days)

gamma <- 0.0035, #Strength of density dependence of worm egg production

kcon <- 0.05, #Aggregation parameter

kin_1 <- 0.043, #Aggregation parameter_1

kin_2 <- 0.028, #Aggregation parameter_2

kin_3 <- 0.018, #Aggregation parameter_3

kin_4 <- 0.011, #Aggregation parameter_4

## No intervention in baseline

## intervention consists of TnT and MDA interventions

## parameters for test and treat(TnT) intervention

## intervention - day

kato_sen <- 0, #Kato test sensitivity

kato_spec <- 0, #Kato test specificity

ProTnT_p <- 0, #Proportion of testing in PSAC by kato per TnT round

ProTnT_c <- 0, #Proportion of testing in SAC by kato per TnT round

ProTnT_a <- 0, #Proportion of testing in Adults by kato per TnT round

h_p <- 0, #drug efficacy for PSAC

h_c <- 0, #drug efficacy for SAC

h_a <- 0, #drug efficacy for Adults

campaigndays_TnT <- 0, #campaigndays_TnT - #duration of TnT intervention (days)

time_interv_TnT <- 0, #time when TnT intervention start (days)

times_per_year_TnT <- 1, #frequency of TnT per year

## parameters for second intervention (nd)

## intervention - day

kato_sen_nd <- 0,

kato_spec_nd <- 0,

ProTnT_p_nd <- 0,

ProTnT_c_nd <- 0,

ProTnT_a_nd <- 0,

campaigndays_TnT_nd <- 0,

time_interv_TnT_nd <- 0,

times_per_year_TnT_nd <- 0.5,

## parameters for MDA

## intervention - day

gMDA_p <- 0, #MDA coverage in PSAC (proportion,between 0-1)

gMDA_c <- 0, #MDA coverage in SAC (proportion,between 0-1)

gMDA_a <- 0, #MDA coverage in adults (proportion,between 0-1)

campaigndays_MDA_p <- 0, #duration of MDA treatment (days)

campaigndays_MDA_c <- 0, #duration of MDA treatment (days)

campaigndays_MDA_a <- 0, #duration of MDA treatment (days)

time_interv_MDA_p <- 0, #time when MDA start in days (days)

time_interv_MDA_c <- 0, #time when MDA start in days (days)

time_interv_MDA_a <- 0, #time when MDA start in days (days)

times_per_year_MDA <- 1, #frequency of MDA per year

## parameters for second intervention (nd)

## intervention - day

gMDA_p_nd <- 0,

gMDA_c_nd <- 0,

gMDA_a_nd <- 0,

campaigndays_MDA_p_nd <- 0,

campaigndays_MDA_c_nd <- 0,

campaigndays_MDA_a_nd <- 0,

time_interv_MDA_p_nd <- 0,

time_interv_MDA_c_nd <- 0,

time_interv_MDA_a_nd <- 0,

times_per_year_MDA_nd <- 0.5)

## Define Helminth transmission Model

Helminth_model <- function (t, state, parameters_1) {

with(as.list(c(state,parameters_1)), {

#Adjust aggregation parameter based on time

k <- if(t > 49){

kin_4

}else if(t > 44){

kin_3

}else if(t > 39){

kin_2

}else if(t > 35){

kin_1

}else {

kcon

}

##mating probability factor (psi) for AS

psi_p <- 1- ((1 + (Mp/k)*(1-exp(-gamma)))/(1 + (Mp/k)*(2-exp(-gamma))))^(k+1)

psi_c <- 1- ((1 + (Mc/k)*(1-exp(-gamma)))/(1 + (Mc/k)*(2-exp(-gamma))))^(k+1)

psi_a <- 1- ((1 + (Ma/k)*(1-exp(-gamma)))/(1 + (Ma/k)*(2-exp(-gamma))))^(k+1)

##mean egg production rate from host with mean worm burden

fp <- Mp/(1 + (Mp/k)*(1-exp(-gamma)))^(k+1)*(psi_p) #for PSAC

fc <- Mc/(1 + (Mc/k)*(1-exp(-gamma)))^(k+1)*(psi_c) #for SAC

fa <- Ma/(1 + (Ma/k)*(1-exp(-gamma)))^(k+1)*(psi_a) #for adults

##estimate prevalence

Pp <- (1-(1+(Mp/k))^-k)

Pc <- (1-(1+(Mc/k))^-k)

Pa <- (1-(1+(Ma/k))^-k)

TotalP <- ((Pp*np)+(Pc*nc)+(Pa*na))/(np+nc+na)

##TnT intervention calculation

##TnT - the number of infected individuals who are detected and #enter the treatment process.

##TnT <- (1-(1+(M/k))^-k)*kato_sen*ProTnT

##For PSAC

TnTp <- if(kato_sen == 0 | ProTnT_p == 0){

0

}else {

(1-(1+(Mp/k))^-k)*kato_sen*ProTnT_p

}

##For SAC

TnTc <- if(kato_sen == 0 | ProTnT_c == 0){

0

}else {

(1-(1+(Mc/k))^-k)*kato_sen*ProTnT_c

}

##For Adults

TnTa <- if(kato_sen == 0 | ProTnT_a == 0){

0

}else {

(1-(1+(Ma/k))^-k)*kato_sen*ProTnT_a

}

##TnTi_rate <- (-log(1-(TnTi*h_i))/campaigndays_TnT),i = PSAC,SAC,Adults

##For PSAC

TnTp_rate <- if(TnTp == 0){

0

}else {

(-log(1-(TnTp*h_p))/campaigndays_TnT)

}

##For SAC

TnTc_rate <- if(TnTc == 0){

0

}else {

(-log(1-(TnTc*h_c))/campaigndays_TnT)

}

##For Adults

TnTa_rate <- if(TnTa == 0){

0

}else {

(-log(1-(TnTa*h_a))/campaigndays_TnT)

}

##The second TnT intervention

##For PSAC

TnTp_nd <- if(kato_sen_nd == 0 | ProTnT_p_nd == 0){

0

}else {

(1-(1+(Mp/k))^-k)*kato_sen_nd*ProTnT_p_nd

}

##For SAC

TnTc_nd <- if(kato_sen_nd == 0 | ProTnT_c_nd == 0){

0

}else {

(1-(1+(Mc/k))^-k)*kato_sen_nd*ProTnT_c_nd

}

##For Adults

TnTa_nd <- if(kato_sen_nd == 0 | ProTnT_a_nd == 0){

0

}else {

(1-(1+(Ma/k))^-k)*kato_sen_nd*ProTnT_a_nd

}

##TnTi_rate <- (-log(1-(TnTi*h_i))/campaigndays_TnT), i = PSAC,SAC,Adults

##For PSAC

TnTp_rate_nd <- if(TnTp_nd == 0){

0

}else {

(-log(1-(TnTp_nd*h_p))/campaigndays_TnT_nd)

}

##For SAC

TnTc_rate_nd <- if(TnTc_nd == 0){

0

}else {

(-log(1-(TnTc_nd*h_c))/campaigndays_TnT_nd)

}

##For Adults

TnTa_rate_nd <- if(TnTa_nd == 0){

0

}else {

(-log(1-(TnTa_nd*h_a))/campaigndays_TnT_nd)

}

##TnT effect in population on specific time

##For PSAC

TnTp_p <- if(t<=35){

0

}else if((t>35)&(t<57)){

(((t>35)&(t<57))>=(T))*((t %% times_per_year_TnT) >=(time_start+time_interv_TnT)) *((t %% times_per_year_TnT)<=time_start+time_interv_TnT+campaigndays_TnT)*TnTp_rate

}else if(t>=57){

((t>=57)>=(T))*((t %% times_per_year_TnT_nd) >=(time_start+time_interv_TnT_nd)) *((t %% times_per_year_TnT_nd)<=time_start+time_interv_TnT_nd+campaigndays_TnT_nd)*TnTp_rate_nd

}

##For SAC

TnTc_c <- if(t<=35){

0

}else if((t>35)&(t<57)){

(((t>35)&(t<57))>=(T))*((t %% times_per_year_TnT) >=(time_start+time_interv_TnT)) *((t %% times_per_year_TnT)<=time_start+time_interv_TnT+campaigndays_TnT)*TnTc_rate

}else if(t>=57){

((t>=57)>=(T))*((t %% times_per_year_TnT_nd) >=(time_start+time_interv_TnT_nd)) *((t %% times_per_year_TnT_nd)<=time_start+time_interv_TnT_nd+campaigndays_TnT_nd)*TnTc_rate_nd

}

##For Adults

TnTa_a <- if(t<=35){

0

}else if((t>35)&(t<57)){

(((t>35)&(t<57))>=(T))*((t %% times_per_year_TnT) >=(time_start+time_interv_TnT)) *((t %% times_per_year_TnT)<=time_start+time_interv_TnT+campaigndays_TnT)*TnTa_rate

}else if(t>=57){

((t>=57)>=(T))*((t %% times_per_year_TnT_nd) >=(time_start+time_interv_TnT_nd)) *((t %% times_per_year_TnT_nd)<=time_start+time_interv_TnT_nd+campaigndays_TnT_nd)*TnTa_rate_nd

}

##MDA treatment intervention calculation

##MDArate_i <- (-log(1-(gMDA_i*h_i))/campaigndays_MDA_i), i = PSAC,SAC,Adults

##For PSAC

MDArate_p <- if(gMDA_p == 0){

0

}else {

(-log(1-(gMDA_p*h_p))/campaigndays_MDA_p)

}

##For PSAC

MDArate_c <- if(gMDA_c == 0){

0

}else {

(-log(1-(gMDA_c*h_c))/campaigndays_MDA_c)

}

##For Adults

MDArate_a <- if(gMDA_a == 0){

0

}else {

(-log(1-(gMDA_a*h_a))/campaigndays_MDA_a)

}

##The second intervention

##MDArate_i <- (-log(1-(gMDA_i*h_i))/campaigndays_MDA_i), i = PSAC,SAC,Adults

##For PSAC

MDArate_p_nd <- if(gMDA_p_nd == 0){

0

}else {

(-log(1-(gMDA_p_nd*h_p))/campaigndays_MDA_p_nd)

}

##For SAC

MDArate_c_nd <- if(gMDA_c_nd == 0){

0

}else {

(-log(1-(gMDA_c_nd*h_c))/campaigndays_MDA_c_nd)

}

##For Adults

MDArate_a_nd <- if(gMDA_a_nd == 0){

0

}else {

(-log(1-(gMDA_a_nd*h_a))/campaigndays_MDA_a_nd)

}

##MDA effect in population on specific time

##For PSAC

MDAcampaign_p <- if (t<=35){

0

}else if((t>35)&(t<57)){

(((t>35)&(t<57))>=(T))*((t %% times_per_year_MDA) >=(time_start+time_interv_MDA_p)) *((t %% times_per_year_MDA)<=time_start+time_interv_MDA_p+campaigndays_MDA_p)*MDArate_p

}else if(t>=57){

((t>=57)>=(T))*((t %% times_per_year_MDA_nd) >=(time_start+time_interv_MDA_p_nd)) *((t %% times_per_year_MDA_nd)<=time_start+time_interv_MDA_p_nd+campaigndays_MDA_p_nd)*MDArate_p_nd

}

##For SAC

MDAcampaign_c <- if (t<=35){

0

}else if((t>35)&(t<57)){

(((t>35)&(t<57))>=(T))*((t %% times_per_year_MDA) >=(time_start+time_interv_MDA_c)) *((t %% times_per_year_MDA)<=time_start+time_interv_MDA_c+campaigndays_MDA_c)*MDArate_c

}else if(t>=57){

((t>=57)>=(T))*((t %% times_per_year_MDA_nd) >=(time_start+time_interv_MDA_c_nd)) *((t %% times_per_year_MDA_nd)<=time_start+time_interv_MDA_c_nd+campaigndays_MDA_c_nd)*MDArate_c_nd

}

##For Adults

MDAcampaign_a <- if (t<=35){

0

}else if((t>35)&(t<57)){

(((t>35)&(t<57))>=(T))*((t %% times_per_year_MDA) >=(time_start+time_interv_MDA_a)) *((t %% times_per_year_MDA)<=time_start+time_interv_MDA_a+campaigndays_MDA_a)*MDArate_a

}else if(t>=57){

((t>=57)>=(T))*((t %% times_per_year_MDA_nd) >=(time_start+time_interv_MDA_a_nd)) *((t %% times_per_year_MDA_nd)<=time_start+time_interv_MDA_a_nd+campaigndays_MDA_a_nd)*MDArate_a_nd

}

##Define differential equations

dMp <- (beta_p*L)-(mu_W*Mp)-(TnTp_p*Mp)-(MDAcampaign_p*Mp)

dMc <- (beta_c*L)-(mu_W*Mc)-(TnTc_c*Mc)-(MDAcampaign_c*Mc)

dMa <- (beta_a*L)-(mu_W*Ma)-(TnTa_a*Ma)-(MDAcampaign_a*Ma)

dL <- (fp*np*alpha_p + fc*nc*alpha_c+ fa*na*alpha_a)-(mu_L+beta_p*np+beta_c*nc+beta_a*na)*L

list (c(dMp, dMc, dMa,dL))

})

}

## Run simulation

baseline_1= ode(y=state, times=times, func=Helminth_model,

parms= parameters_1,method="euler")

## Save data in RDS----

saveRDS(baseline_1,"baseline_1")

Collect.baseline <- readRDS(file ="baseline_1")

## create dataframe-data.frame(out_baseline)

baseline_1dataframe <-data.frame(Collect.baseline)

# End of Supplementary Material
